# Synthesis and structural insights of bis(2-methoxy-6-{[(2-methylpropyl)imino]methyl}phenolato) nickel (II) complex through DFT and docking investigations

**DOI:** 10.1038/s41598-025-85465-6

**Published:** 2025-01-11

**Authors:** K. Jayachandiran, Sv. Esha, M. Savitha Lakshmi, S. Mahalakshmi, S. Arockiasamy

**Affiliations:** Chemistry Department, School of Advanced Sciences, Vellore Institute of Technology-Chennai campus, Chennai, 600127 India

**Keywords:** Schiff base, Nickel complex, Synthesis, Crystal data, Biological activity, DFT, Docking study, Biochemistry, Chemical biology, Computational biology and bioinformatics, Drug discovery, Chemistry

## Abstract

Nickel complexes are a potential candidate for antibacterial and antifungal activity. A new Ni (II) complex, bis(2-methoxy-6-{[(2-methylpropyl)imino]methyl}phenolato)nickel (II) (**2**), was synthesised by reacting, bis(3-methoxy-salicylaldehyde)nickel (II) (**1**) with isobutylamine. It was characterised by single crystal X-ray diffraction (ScXRD), UV-Vis, NMR, IR, mass spectrometry, and thermogravimetry (TG) to study its structure and physico-chemical properties. The ScXRD showed a square planar geometry, and monoclinic crystal system with a space group P21/n. The TG analysis revealed its thermal durability pre and post-melting up to 225 ^o^C with a weight loss of only 2%. The optimized molecular structure, energy gap between HOMO and LUMO, and intermolecular interactions were studied by computational methods. The microbial activity evaluation showed significant anti-bacterial activity against *E. coli* and *S. aureus* when the concentration exceeded 40 µg/mL, and a prominent anti-fungal activity over *C. albicans* and *C. tropicalis* above 30 µg/mL. The values of minimum inhibitory concentration (MIC) for bacteria (MIB) and fungi (MIF) implied its potential to inhibit the growth of microbes. Docking studies revealed that the molecule binds well with proteins such as PDB: 2W9H for Dihydrofolate Reductase of *S.aureus* as shown by its binding energy of -8.62 kcal.mol^− 1^.

**Supplementary Information:**

The online version contains supplementary material available at 10.1038/s41598-025-85465-6.

## Introduction

In the long history of coordination compounds, Schiff bases are predominantly famous for their significant role as ligands which find extensive and exceptional applications in various fields^1,2^. The -HC = N imine bond, consisting of an electrophilic carbon and a nucleophilic nitrogen facilitates binding possibilities with various nucleophiles and electrophiles^3^. This characteristic flexibility is the key to inhibiting specific diseases, enzymes, or DNA replication. The azomethine linkage [-C = N] provides great versatility in creating Schiff base ligands with higher and selective metal ion coordination capabilities^4^ enabling it for distinct function in biological activities^5–8^.

Owing to their prominence and usability in synthetic chemistry, Schiff base complexes are mostly biologically active and are of great pharmaceutical significance^9,10^. Schiff Base complexes exhibit a wide array of exceptional biological activities including anti-tuberculosis^11^, anti-platelet^12^, anti-diabetic^13^, anti-arthritic^14^, anti-oxidant^15^, anti-inflammatory^16^, anti-cancer^17^, anti-viral^18^, anti-malarial^19^, analgesic^20^, anti-fungal^21^, anti-bacterial^22^, and anti-microbial^23^ capabilities. Also, Schiff base complexes find extensive application in industries^24^, degradation of dyes^25^, polymerization^26^, catalysis^27^, and precursors for chemical vapour deposition^28^.

For all biological activities, metal traces are essential because about 30 to 40% of all proteins, like metalloenzymes, need metallic cofactors such as Iron (Fe), Zinc (Zn), Nickel (Ni), or Manganese (Mn) for precise folding into a functional 3-D structure^5,29^. Due to their outstanding physiochemical properties, the significance of Schiff base complexes in coordination chemistry is well documented. Their properties and functions vary depending on the metal type and the functional groups on the Schiff base ligand moiety. Schiff bases derived from ortho-vanillin, the chief flavour and odour compound of the vanilla plant, along with salicylaldehyde, finds extensive applications as bidentate or multidentate ligands^30^. Ortho-vanillin is widely researched in the field of medicinal chemistry due to its exceptional biological activities^31^. Also, Schiff bases originating from ortho-vanillin were proven to interact with DNA and display a variety of biological activities such as anti-microbial, anti-fungal, anti-oxidant, and anti-cancer^32^. Isobutyl amine, a predominant amine component of natural amides has shown biological activity as an analgesic^33^. Ni (II) complexes incorporating Schiff base consisting of ortho-vanillin and a variety of amines have shown enhanced biological activities^34–37^ and have proven to be more potent than Cu (II) at certain combinations^38^.

Inspired by the results of proven research, provided in the literature regarding the exceptional biological activities of Ni (II) metal complexes incorporating Schiff base, ortho-vanillin, and isobutyl amine, in this work, we intended to synthesize bis (2-methoxy-6-{[(2-methyl propyl)imino]methyl}phenolato) nickel (II) (**2**) complex and elaborate its complete study via series of characterization techniques, in anticipation to reveal a commendable biological activity. The thermogravimetric analysis (TGA) of a complex is essential to determine its shelf-life (especially for potential therapeutic applicants)^39^, while the spectral study confirms the elements and functional groups present along with their purity^40^. The crystallographic studies depict the structure’s geometry to facilitate a better understanding of their coordination sphere^41^. The computational-based drug design tool of molecular docking is useful in evaluating the binding energy of proposed drug molecules with respective proteins and reducing free energy by optimizing the conformation of the drug and protein concerning each other^42^.

Here, we present, elemental, single crystal X-ray diffraction, thermogravimetric analysis (TGA), Fourier transformed infrared spectral (FTIR), UV-vis, mass spectrometry, and nuclear magnetic resonance (NMR) analysis of a new nickel complex. We also report the biological evaluation of minimum inhibition concentration (MIC), Minimum bactericidal concentration (MBC), and minimum fungicidal concentration (MFC). Computational studies such as Density functional theory (DFT), Hirshfeld surface analysis (HAS), and molecular docking were carried out to support the experimental data.

### Synthesis of nickel complexes 1 and 2

**Fig. 1:**
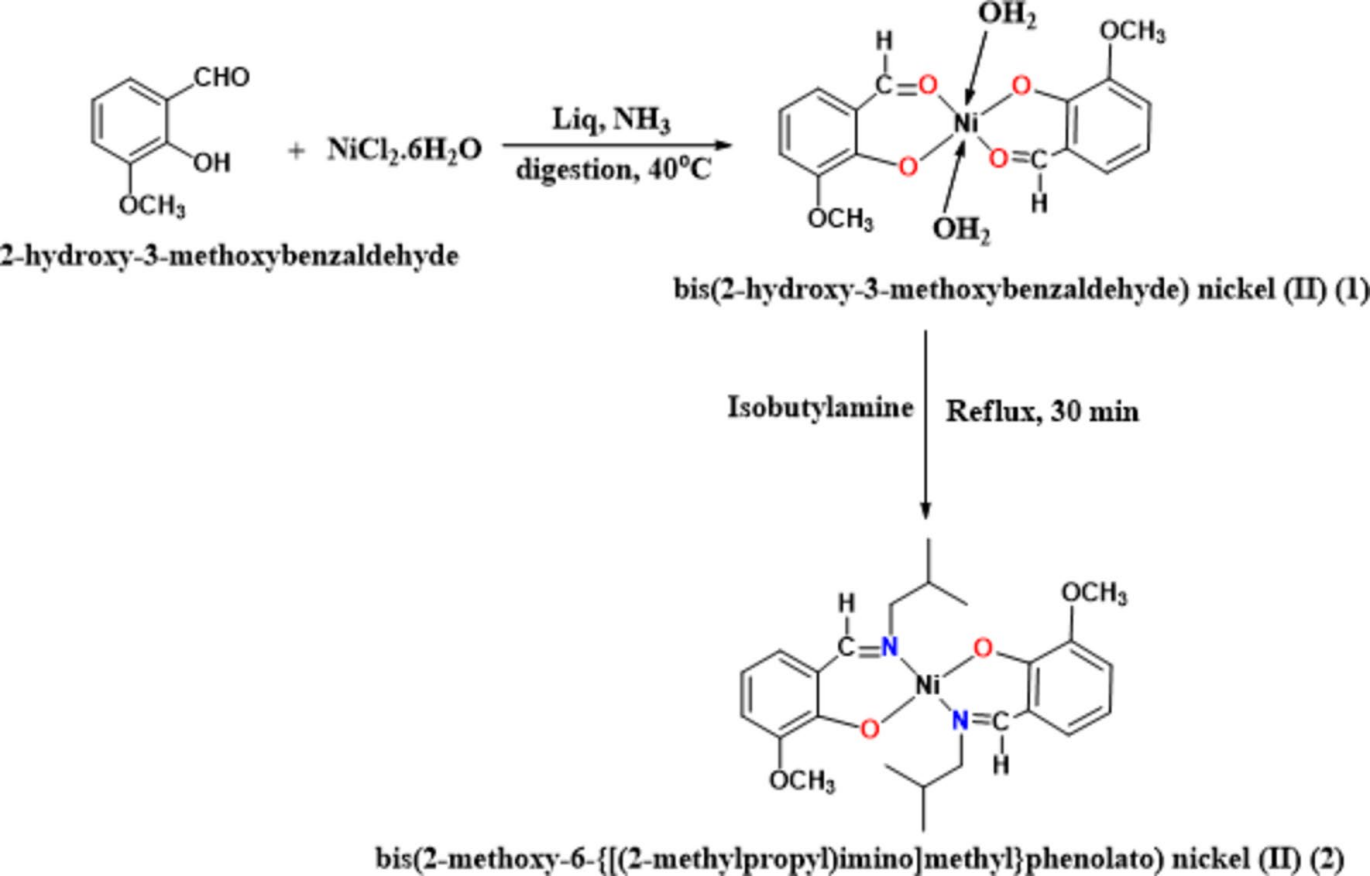
Synthesis of nickel complex **2** using the parent complex **1**.

The chemicals, 3-methoxysalicylaldehyde (Merck, purity, 99%), NiCl_2_.6H_2_O (Merck, purity > 98%), isobutylamine (Merck, purity, 99%), the solvents such as methanol, ethanol, and liq. Ammonia (AVRA, INIDA) was purchased and used as such without any purification. Owing to the unsuccessful attempt to synthesise the Schiff base ligand by condensing 3-methoxy-salicylaldehyde and isobutylamine, the following method reported by our group earlier was used for preparing the nickel complex^39^. A parent compound, bis(3-methoxy-salicylaldehyde)nickel (II) (**1**) was initially synthesised by mixing 1.5 g (0.006 mol) of NiCl_2_.6H_2_O in 10 cm^3^ of water and 2.0 g (0.012 mol) of 3-methoxy-salicylaldehyde in 10 cm^3^ of ethanol under continuous stirring maintaining a 1:2 mol ratio. The reaction mixture was then subjected to dropwise addition of liq. ammonia (2 cm^3^) while closely monitoring the formation of a fluorescent green precipitate (Fig. 1). The resultant precipitate was digested at 60 ºC over a water bath for 30 min. After digestion, the precipitate was filtered, washed thoroughly with water, and dried under vacuum overnight.

For the synthesis of **2**, a gradual addition of 2 mL of isobutylamine to a warm suspension containing 0.5–1.0 g of bis(3-methoxy-salicylaldehyde) nickel (II) (**1**) in aqueous methanol was meticulously carried out at 40 ºC over a water bath until the solution turned clear olive green in colour. Subsequently, the resulting reaction mixture was transferred into an RB flask and refluxed for 30 min. The resulting olive green crystals were filtered, washed with ethanol, and dried under vacuum overnight. The crystals were recrystallised repeatedly using methanol to enhance their purity and used for crystallography analysis. Complex **1** was an intermediate compound in the preparation of complex **2** and henceforth all the analysis and discussion corresponding to complex **2** only.

Complex **2** : M = 471.1 g.mol^− 1^, Ni: 13.2%), mp:179–182 ^o^C. Formula: C24H32N2O4Ni, calcld. (%): C, 61.17; H, 6.85; N, 5.94. Found (%): C, 60.01; H, 6.91; N, 6.80. *δ*_H_, ^1^H NMR (25 ◦C, 500 MHz, CDCl3): 8.78 (s, 1H, N = HC-), 7.26 (due to CDCl_3_), 6.76–6.67 (m, 3H, C-H aryl), 3.72 (s, 3H, -OCH_3_), 3.63 (s, 2 H, -CH_2_- from iso-butyl), 2.48–2.40 (m, 1H, -CH from iso-butyl), 1.58 (residual water), 1.0–0.99 (s, 6 H, two CH_3_ from iso-butyl). *δ*_C_, ^13^C NMR (25 ◦C, 500 MHz, CDCl_3_): 163.0 (s, –CH–C_6_H_5_), 154.2-151.5 (s, –C_6_H_5_), 123.1–111.8 (m, C_6_H_5_), 65.5 (s, -N-CH_2_-CH-(CH_3_)_2_), 55.2 (s, (C-O-CH_3_), 30.7 (s, N– CH_2_-CH-(CH_3_)_2_). CDCl_3_ peaks at 76.82–77.33. Cell parameters, a = 6.2835(5) A, b = 17.4797(14) A, c = 21.2542(17) A, α = 90 ^o^, β = 93.779(3) ^o^, γ = 90^o^, Z = 4, density = 1.344 Mg/m^3^.

### Physicochemical characterisations

The crystal structure analysis of **2** was done by using Bruker APEX 2 CCD diffractometer equipped with ω and ϕ scan mode with Mo Kα (λ = 0.71073Å) at T = 298(2) K. The Crystal structure was solved by SHELXL 2019/3 using the direct method and the refinement was performed by full matrix least squares of F^2^^38^. SAINT and SADABS programs were used to correct the data for Lorentzian, polarisation, and absorption effects. EURO VECTOR EA 3000 was used for elemental analysis. The UV-vis spectrum was acquired on a PE UV SUBTECH SPECTRUM ASCII PEDS 4.00 instrument within a targeted wavelength range of 200–700 nm using a quartz cuvette with a 1 cm path length. A 100 ppm of methanolic solution was used for UV-vis measurements. Baseline correction was meticulously performed using the corresponding solvent blank. Infrared (IR) analysis was performed using a Nicolet iS10-FTIR spectrometer in the wavelength range 4000 to 450 cm^− 1^. NMR and mass spectral data were acquired using Bruker Avance III 500 spectrometer and Waters Xevo G2-XS Q-Top mass spectrometer 6200 series respectively. Thermal analysis was carried out using a Hitachi (model 7200) TG/DTA by passing a purge gas of high pure inert N_2_ of 200 mL/min at a heating rate of 10 ^o^C/min. The powder X-ray diffraction pattern was recorded using the model D8 advance BRUKER in the 2θ range of 10–90° using CuKα (1.5406 Å) radiation. To obtain the theoretical electronic spectra using the polarisable continuum solvation model (PCM), time-dependent density functional theory (TD-DFT) calculation was performed in methanol solvent.

### Antimicrobial studies

The antibacterial and antifungal activities of complex **2** were evaluated using the agar well diffusion method. For antibacterial testing, concentrations of 10 µg/mL, 20 µg/mL, 30 µg/mL, and 40 µg/mL prepared in DMSO (purity 99%, AVRA) were utilized against *Escherichia coli* (ATCC25922) and *Staphylococcus aureus* (ATCC25923). Cultures of the test organisms were propagated in sterile Nutrient broth and incubated for 24–48 h. Further, sterile Mueller-Hinton Agar plates were prepared and inoculated with 0.1% inoculum suspensions of the test organisms. Wells with a diameter of 6 mm were created on the agar surface, and each concentration was added to the wells (approximately 20 µL). The plates were then incubated at 37 ºC for 24 h, after which the antibacterial activity was assessed by measuring the diameter of the inhabitation zones around the wells. Results were compared with streptomycin-positive and DMSO-negative controls.

Imidazole and DMSO were used as the positive and negative controls for antifungal activity evaluation against *Candida albicans* (ATCC10231) and *Candida tropicalis* (ATCC4563). Similar to the antibacterial testing, the test organisms were cultured in sterile nutrient broth and incubated for 25–48 h. Sterile Mueller-Hinton agar plates were prepared and inoculated with 0.1% inoculum suspensions of *Candida albicans* and Candida tropicalis. Wells were created on the agar surface, and each sample was added to the wells. The plates were then incubated at 37 ºC for 24 h, after which the antifungal activity was determined by measuring the diameter of the inhibition zones around the wells. Results were compared with the positive and negative controls. The minimum inhibitory concentration (MIC) of the compound was determined to be the lowest dilution that prevented the growth of each test bacteria (*Escherichia coli* and *Staphylococcus aureus*) and fungi (*Candida albicans* and *Candida tropicalis*) as reported earlier^43^.

### Computational study

Density functional theory (DFT) calculations on **2** were performed by adopting Gaussian 09 software^44^. The geometry of the complex was constructed and the optimization was carried out in the gas phase with the help of hybrid functional (B3LYP) and 6–311 g(d, p) and LANL2DZ basis sets for nontransition metal elements and transition metal respectively. The LANL2DZ basis set includes pseudopotentials for the core electrons of the metal. The quantum chemical descriptors and the MEP surface were obtained. Hirshfeld surface and corresponding 2D fingerprint plots were generated for the crystal surface using Crystal Explorer 21.5^45^. The crystallographic information file acquired from the crystallography experimental measurements was used as the input file. Non-covalent interactions (NCI) were analyzed using the reduced density gradient (RDG) method with Multiwfn software^46^ and visual molecular dynamics (VMD)^47^.

The optimized geometry of **2** was utilized for the docking study. The targets chosen for this analysis were *Escherichia coli* Gyrase B (PDB ID: 6F86), *Staphylococcus aureus* Dihydrofolate Reductase (PDB ID: 2W9H), *Candida albicans* agglutinin-like Als9 protein (PDB ID: 2YLH), and *Candida tropicalis* PCIF1_WW domain-containing protein (PDB ID: 8BH9). The crystal structures of the target were obtained from an online source, a protein data bank at (https://www.rcsb.org). The downloaded proteins were cleaned by removing co-crystallized substrates and water molecules. The docking study was carried out using docking programs like AutoDock Tools (ADT) version 1.5.6 and AutoDock version 4.2.6^48^. Standard docking parameters were applied and a grid box (100 × 100 × 100, spacing 1Å) was constructed. An efficient and durable Lamarckian Genetic Algorithm (LGA) was adopted in this study. Discovery Studio software was used to visualize the interactions between the amino acids and drug molecules.

## Results and discussion

### UV-Vis spectral analysis

The UV-Vis spectrum of **2** as shown in Fig. S1, revealed two prominent peaks with substantial absorbance at 236 nm and 260 nm, suggesting the presence of chromophores such as aromatic rings or conjugated double bonds. These peaks assuredly indicate the existence of chromophores within the complex that exhibit the strongest light absorption at these specific wavelengths. Aromatic benzene rings and conjugated double bonds^49^ can participate in π---π* transitions, potentially leading to the observed peaks. A comparatively lower intensity band of absorption (a non-sharp, diffused peak) is observed at 386 nm due to the n—π* transition involving the non-bonding electrons on the nitrogen atom of the azomethine moiety which occurs at longer wavelengths than π---π* transitions^50^. The simulated absorption spectrum (200–1000 nm) is shown in Fig. S1, and the calculated data are given in Table S1. The simulated absorption spectrum is in acceptable agreement with the experimental absorption spectrum. The calculated transition corresponding to the experimentally observed band is assigned based on the computed transition’s energy and the oscillator strength (f). The experimentally observed bands at 236, 260, and 386 nm correspond to 249, 264, and 391 nm of the simulated transition, respectively. The bands at 249 and 264 nm are intense bands with ligand-to-metal charge transfer (LMCT) character and a low intense band is observed at 391 nm with metal-to-ligand charge transfer (MLCT) character. The 249, 264, and 391 nm bands are produced with HOMO-6 ◊ LUMO + 2 (49%), HOMO-6 ◊ LUMO (46%), and HOMO-3 ◊ LUMO + 2 (32%), and HOMO ◊ LUMO + 1 (95%), respectively.

### FTIR analysis

The aldimine C–H asymmetric stretching appears at 2958 cm^–1^ while the azomethine (-C = N) stretching appears (Fig. S2) as a strong band in the region 1607 cm^–1^. The presence of this peak confirms the formation of the C = N bond between the carbonyl group of the salicylaldehyde and the amine group. The broad peak at υ(C-H), 2860–3003 cm^–1^ signifies the stretching vibrations of aliphatic C-H bonds, likely from the isobutyl group of the complex. The aromatic υ(C = C) stretching vibration bands are observed at 1601, and 1458 cm^–1^ within the complex structure, potentially arising from the salicylaldehyde moiety. The peak at 646 cm^–1^ corresponds to the υ(Ni-O) stretching vibration, confirming the formation of a bond between the nickel and the oxygen atom of the deprotonated salicylaldehyde moiety. This peak at 539 cm^–1^ is attributed to the υ(Ni-N) stretching vibration between the nickel (Ni) atom and the nitrogen (N) atom of the Schiff base ligand.

### 1H-NMR analysis

The ^1^H NMR spectrum (Fig. S3a) revealed a broad peak at 8.78 ppm. This chemical shift corresponds to a proton on the Schiff base ligand’s azomethine moiety (-CH = N) coordinated with the nickel metal. The peaks between 6.76 ppm and 6.67 ppm likely represent multiple aromatic protons (Aryl-H). Furthermore, the peak at 3.72 ppm suggests the presence of protons associated with methoxy groups (-OCH_3_)^51^. The δ range between 2.48 and 2.40 ppm encompasses multiple peaks, demonstrating aliphatic protons (CH_2_-, CH_3_-) in the molecule, originating from the isobutyl chain. Finally, the combined peaks at 1.0 and 0.99 ppm likely represent equivalent methyl protons (CH_3_-) in a similar environment, possibly part of the two isobutyl groups (-CH_2_-CH(CH_3_)_2_).

### 13C NMR analysis

The ^13^C NMR spectrum (Fig. S3b) provides further structural information and confirms the spectral data from the H^1^ NMR. The peak observed at 163.0 ppm is characteristic of azomethine carbon (-C = N) in the Schiff base ligand. The peaks between 154.2 ppm and 151.5 ppm correspond to carbon atoms within aromatic rings^52^. The range spanning 123.1 to 111.8 ppm typically encompasses aromatic carbons (Aromatic-C) and/or carbon atoms positioned near electronegative groups (e.g., C-O in methoxy groups) in the complex. The peaks observed between 77.3 ppm CDCl_3_ typically resonate around 77 ppm, and its presence can sometimes obscure signals from other carbons in this region. The peak at 65.5 ppm includes carbon atoms from aliphatic chains (C-C) or carbons near other heteroatoms (e.g., oxygen) in the molecule. The prominent peak at 55.2 ppm aligns well with the presence of a methoxy group carbon (-OCH_3_). Finally, the peak at 30.7 ppm falls within the range expected for aliphatic carbon atoms (C-C) in the molecule due to the isobutyl side chain.

### Mass spectral analysis

The hypothesized molecular formula and stoichiometry were evaluated using weight and m/z measurements. The nickel complex showed a cluster peak (Fig. S4a) associated with the isotopic abundance (Fig. S4b) of natural nickel at *m/z* = 471.18 (estimated *m/z* = 470) due to molecular ions (M + H)^+^, corresponding to the monomeric mass of the complex in a solid state. The theoretical isotopic distribution of complex **2** is given in Fig. S4c. Additional peaks found include *m/z* = 411 indicating a possible loss of two methoxy (-OCH_3_) from the molecular ion peak. The peak at *m/z* = 386 could be assigned to the loss of two hydrocarbon moieties, [-(HC-(CH_3_))_2_]. As reported earlier, the peaks at 619 and 544 could be attributed to the formation of associated structures in this type of complex^53^.

### Thermogravimteric analysis (TGA)

**Fig. 2:**
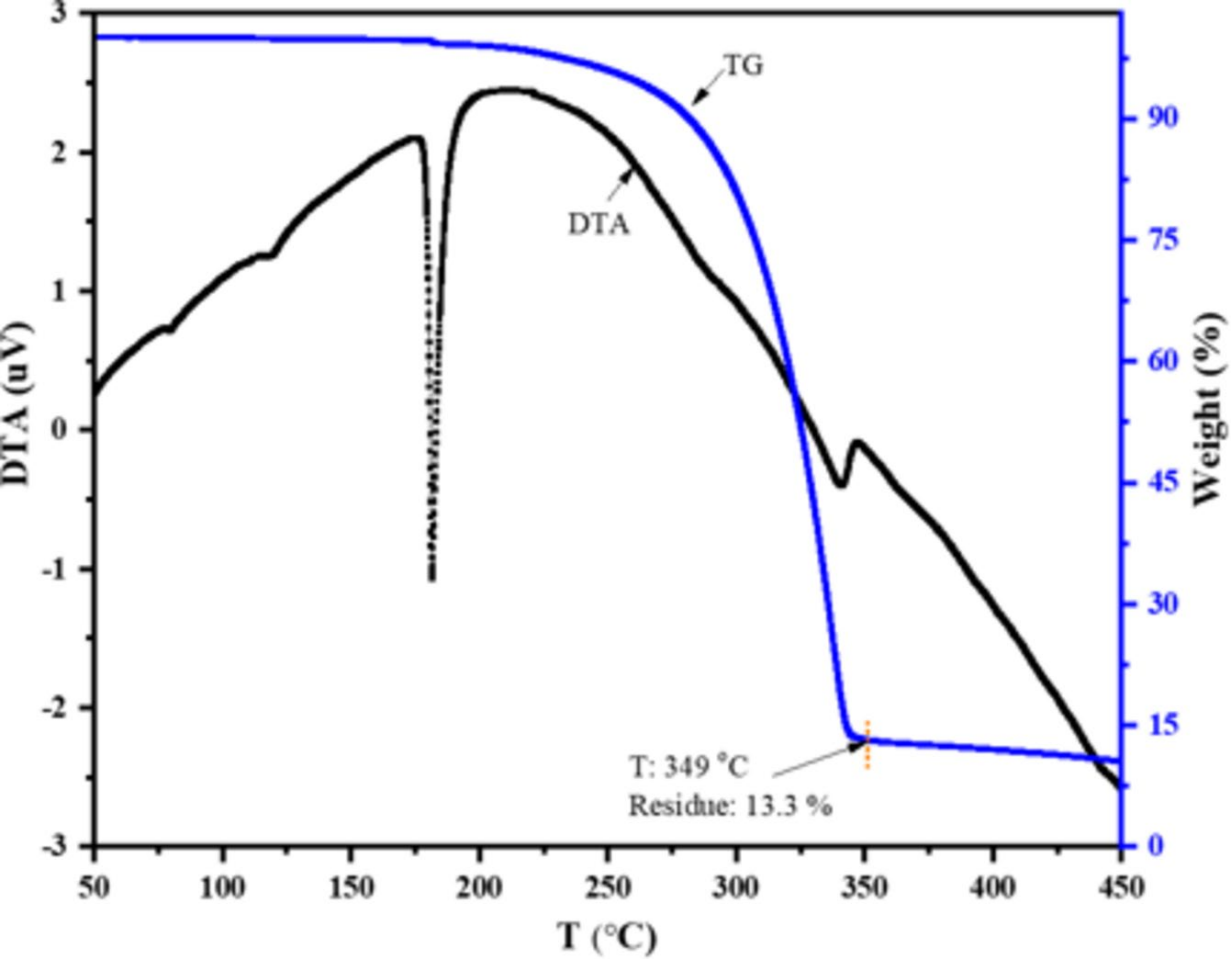
TG/DTA pattern of **2** recorded at a heating rate of 10 °C/min in N_2_ gas purge rate of 200 mL/min.

The non-isothermal TG/DTA (Fig. 2) profile shows, no endothermic peak below 100 ºC in its DTA, indicating the absence of adsorbed/coordinated water molecules. Between 179 to182 ºC, the DTA curve shows a sharp endothermic peak, indicating the melting transition. The nickel complex shows good thermal stability of its solid phase between 40 and 179 ºC, followed by liquid phase stability from 182 to 225 ºC before decomposing. The weight loss is only 2%, between 40 and 225 ºC, confirming the compound’s thermal durability pre/post-melting. The weight reduction in the TG curve after 225 ºC was rather rapid due to the progressive organic moiety elimination. This leads to a greenish-black NiO/C residue of 13.3% at 349 ºC in a single-step decomposition consistent with previous observations of comparable complexes under inert N_2_ atmospheres^39,54^. This step was followed by a sluggish weight loss leading to a final residue of 10.5% at 450 ^o^C.

### Single crystal XRD analysis

**Fig. 3:**
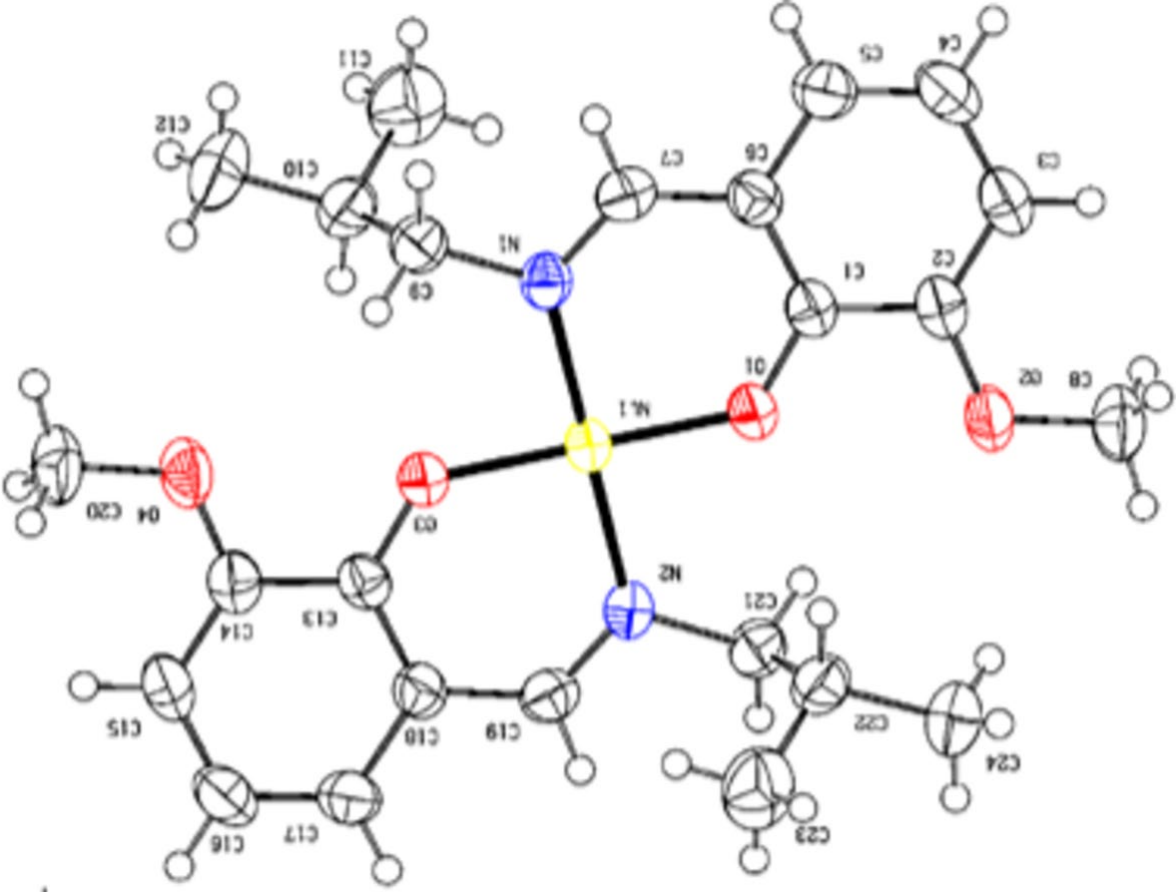
ORTEP of the nickel complex **2** drawn at 50% probability.

The crystallographic data summary of the nickel complex is given in Table S2. Table S3 shows selected bond length (Ǻ) and angles (º). The ORTEP of the complex is shown in Fig. 3. In the crystal structure, the bidentate ligand is coordinated to nickel through one phenolic-O and one imine-N donor site, which are positioned in trans-positions within the nickel coordination sphere. Notably, the nickel atom lies on the inversion center (NiN_2_O_2_) forming a square planar geometry. The coordination sphere’s planar geometry is attributed to the sp^2^-hybridization and center of symmetry of the two nitrogen atoms around nickel, resulting in a total of 360 º bond angle (Table S3). When compared to the parent compound without nitrogen coordination, Ni(1)-O(2) bond length decreases from 2.04 to 1.928(3) Ǻ, [Ni(1)-N(1) in this complex], resulting in increased covalent character. The Ni(1)-N(1)-C(9) bond angle is 122.1(2) º, and the rotational motion of the isobutyl group prevents water molecules from coordinating with nickel, resulting in a coordinatively saturated nickel complex^28^. The basal plane NiN2O2 is formed by coordinating two nitrogen atoms of azomethine nitrogen (-HC = N) and two phenolic oxygen to nickel. The arrangement results in two six-membered chelate rings [Ni(1)-N(1)-C(7)-C(6)-C(1)-O(1)] and [Ni(1)-N(2)-C(19)-C(18)-C(13)-O(3)], as seen in ORTEP Diagram^55^ which provides additional stability. The packing pattern (Fig. S5) seen along the *a*-axis indicates that crystal packing has no significant contact. The absence of intermolecular hydrogen bonds and π…π interactions within van der Waals radii indicates a crystal packing configuration dominated by weak interactions. The ligand-molecular arrangement and antiparallel alignment of molecular planes generate a one-dimensional chain parallel to the crystallographic plane as shown in Fig. S6. Examining the nickel complex 2 with powder X-ray diffraction and comparing it with the theoretical XRD patterns (Fig. S7) confirmed the purity and crystallinity as no other phases could be found.

### DFT calculations

#### Geometry optimization and Frontier molecular orbital analysis

**Fig. 4:**
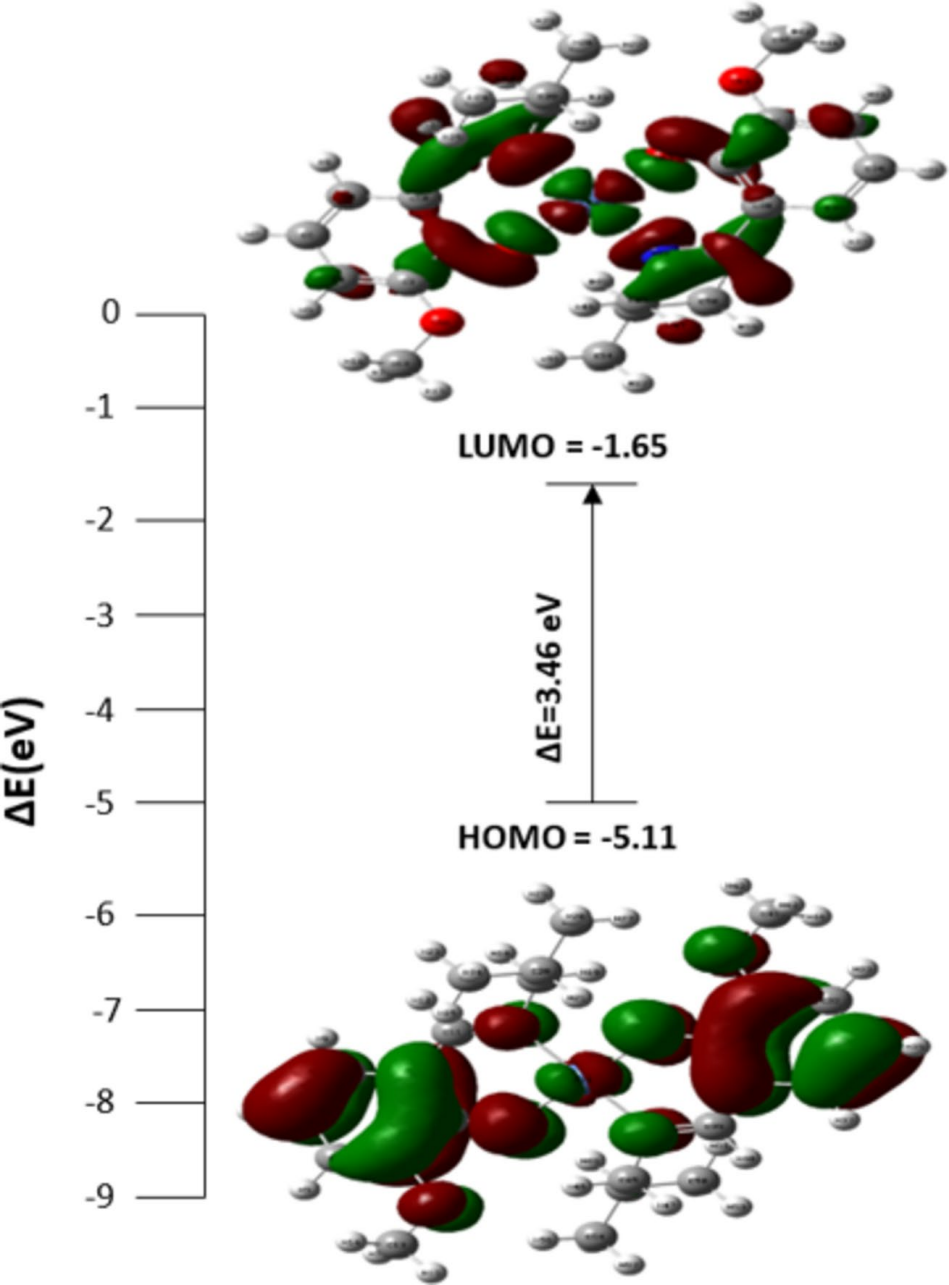
HOMO-LUMO energies and energy gap of **2**.

The geometry optimisation of the nickel complex revealed it to possess square planar geometry around the nickel center as shown in Fig. S8. The vibrational frequencies were analysed to confirm the minimum stationary point without negative frequencies. The frontier molecular orbital (FMO) energies impart information on the chemical reactivity, stability, and electronic properties^56^. The highest occupied molecular orbital (HOMO) and the lowest unoccupied molecular orbital (LUMO) energies are used to obtain different properties like the energy gap, ionization potential, electron affinity, chemical potential, hardness and softness, electrophilicity and nucleophilicity as shown in Table S4. The electron density distribution and energies of the FMO of nickel complex complexes are shown in Fig. 4. The HOMO functions as the electron donor. At the same time, the LUMO serves as the electron acceptor. The energy gap between the HOMO and LUMO helps to understand the chemical stability, the larger energy gap is highly stable and less polarised. The low energy gap is favorable for chemical reactivity in biological applications owing to the ability to carry out efficient charge transfer interaction. The energy gap of the nickel complex is 3.46 eV and the electron density is distributed over the nitrogen, oxygen, and Ni in the case of both HOMO and LUMO. The negative chemical potential (µ = – 3.38 eV) indicates the stability of the complex. Complex **2** has a high electrophilicity (ω = 3.3 eV) and low chemical potential attributed to its electrophilic activity.

### Molecular electrostatic potential

The Molecular electrostatic potential (MEP) map of the **2** is shown in Fig. S9. The MEP map depicts distinct colors representing the nucleophilic and electrophilic activity of the molecule. The negative region, which is an electron-rich site, is shown in red colour is susceptible to electrophilic assault. The blue colour represents the electron-deficient positive area, susceptible to nucleophilic attack. The negative areas are concentrated around oxygen (O) atoms in the nickel complex due to the electron abundance in these areas leading to the possibility of an electrophilic attack. The positive regions in blue may act as H-bond donors in protein-substrate intermolecular interactions.

### Hirshfeld surface analysis (HAS)

The Hirshfeld surface helps to visualize and quantify the intermolecular interaction of the crystals^57,58^. Fig. S10 shows the Hirshfeld surface of **2** with d_norm_ mapped with neighboring molecules. The Hirshfeld surface in 3D d_norm_ was obtained and mapped over a range of -0.0251 to 1.5363 Å. The red spots indicate the interconnects present in intermolecular interaction. The blue regions suggest it is too long to interact with the neighboring atoms. The notable contacts are C…H and H…H over the red spots. The globularity is 0.725 and asphericity is 0.138 indicating that the molecule is moderately spherical with slight anisotropy leading to stable crystals with unique interaction patterns.

**Fig. 5:**
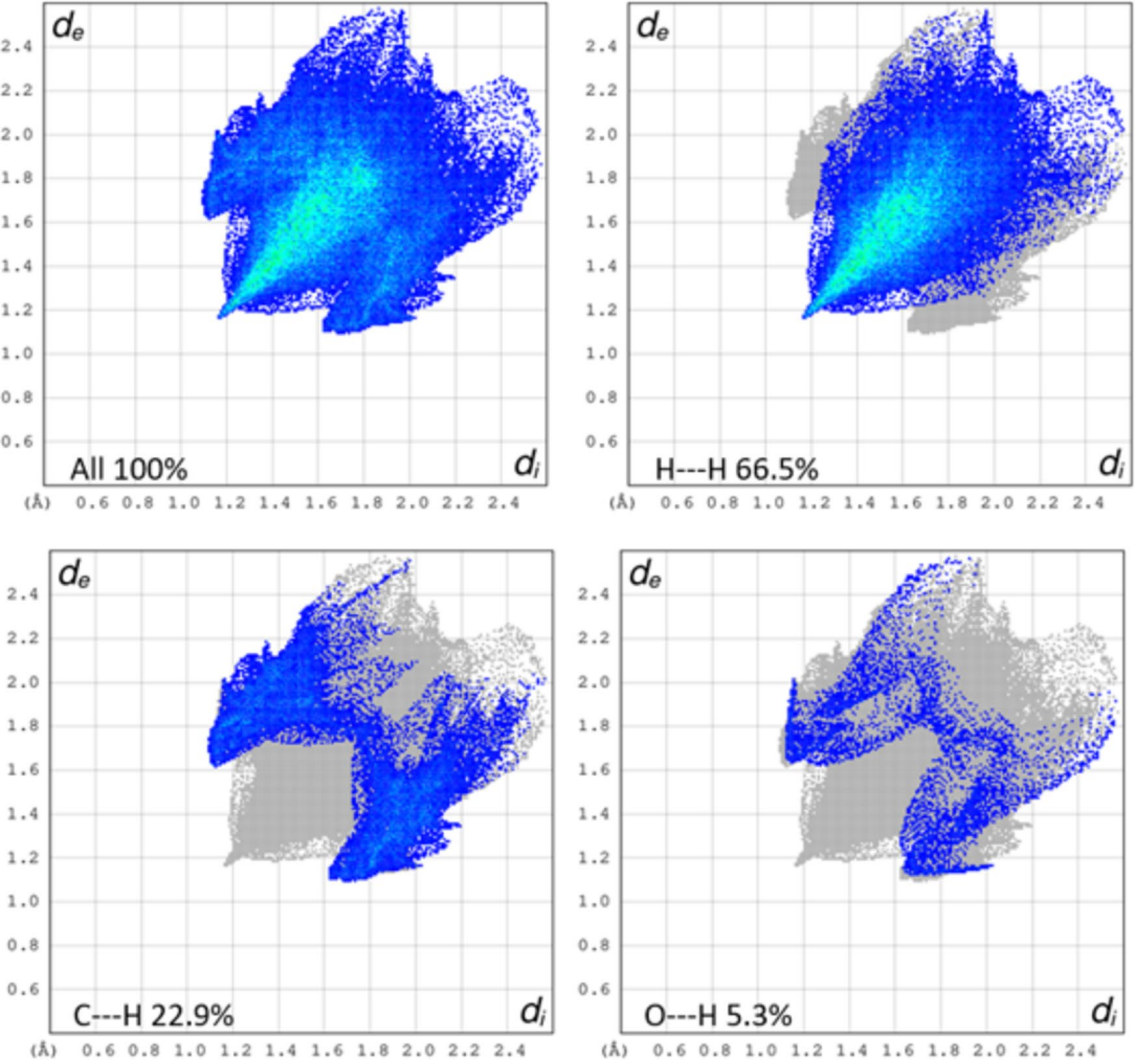
2D fingerprint plots of intermolecular interaction along with percentage contribution.

The fingerprint plots represent the percentage contribution of different intermolecular interactions. Figure 5 displays the significant intermolecular contacts contributing to the Hirshfeld surface. The blue region indicates the assigned reciprocal contacts and the grey shadow suggests the outline of the original fingerprint plot. The *d*_*e*_ and *d*_*i*_ are the distances from the Hirshfeld surface to the nearest atoms outside and inside the surface. The H-H intermolecular interaction accounts for 66.5% at d_i_=d_e_=1.19 Å. The C-H/H-C 22.9% and O-H/H-O 5.3% intermolecular contacts showed a butterfly fingerprint with two broad peaks at d_i_+d_e_ 2.8 Å and d_i_+d_e_ 2.8 Å respectively.

### Non-covalent interactions (NCI)

**Fig. 6:**
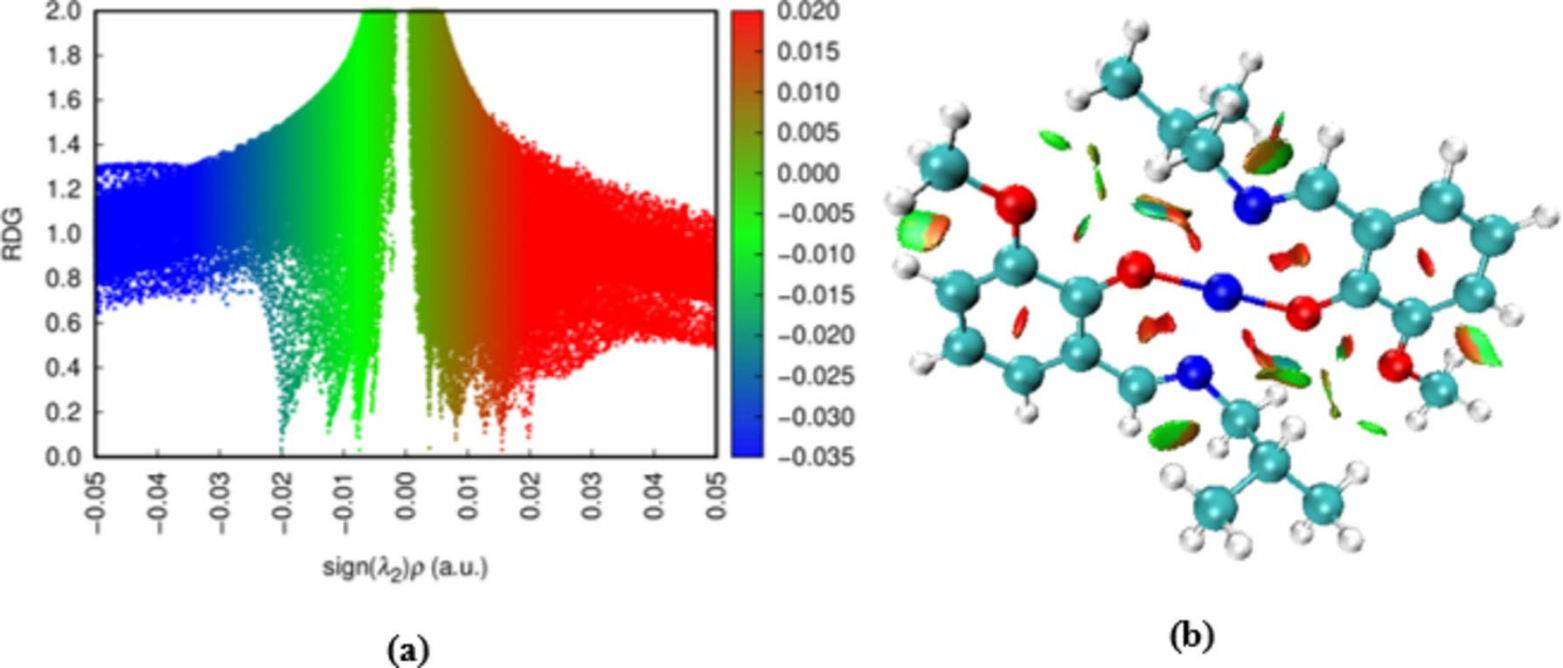
Non-covalent interactions, (**a**) Two-dimensional RDG vs. sign (λ_2_)ρ scatter plot, and (**b**) NCI isosurface plot index.

The NCI plot index provides quantitative and qualitative information about electron densities^59^. This index locates the region where the reduced density gradient approaches zero and well-defined troughs are formed as shown in Fig. 6a. Many well-defined troughs are observed. The red color indicates the repulsive interaction due to the steric clash between the electrons. The green color represents attractive interactions, such as weak van der Waals forces, and the blue colour indicates strong hydrogen bonding^60^. The intensity of the color signifies the strength of the interaction with more intense color implying stronger interaction. The location of these interactions can be visualized as shown in Fig. 6b. The red isosurfaces in the center of the aromatic ring indicate the ring closure interactions and the multi-colored (red/green) indicate van der Waals repulsive and attractive interaction^61^.

### Antibacterial and antifungal studies

In examining antibacterial activity (Table S5) against *Escherichia coli* and *Staphylococcus aureus*, the nickel complex showed equivalent inhibitory zones to the positive control antifungal drug, streptomycin. The disc diffusion assay data demonstrated an inhibition zone of 20 mm for both species. In evaluating antifungal potential against *Candida tropicalis* and *Candida albicans*, the nickel complex demonstrated comparable inhibition zones to the positive control antifungal agent, imidazole. Disc diffusion assay data revealed inhibition zones ranging from 19 to 22 mm, indicating significant antifungal activity.

The antibacterial and antifungal efficacy of the nickel complex was assessed by the determination of minimum inhibitory concentration (MIC)^62^ (Table S6). Among the concentrations tested, 10 µg/mL failed to show activity against *Escherichia coli* and *Staphylococcus aureus*. Conversely, the 20 µg/mL concentration generated 9 and 12 mm inhibition zones against both bacterial strains. Improved antibacterial activity was noted at the 30 µg/mL concentration, yielding inhibition zones of approximately 13 and 14 mm against *Escherichia coli* and *Staphylococcus aureus*, respectively. The 40 µg/mL concentration generated inhibition zones measuring 18 mm against both the antibacterial strains. Also, the Fungi, *Candida albicans*, and *Candida tropicalis* failed to exhibit activity in the concentration of 10 µg/mL, but at the concentration of 20 µg/mL generated inhabitation zones at 11 mm and 15 mm against both fungal strains. Also at 30 µg/mL concentration, these antifungal activities show a 13 mm and 18 mm zone of inhibition^63^. Finally, in the 40 µg/mL, the inhibition zones of 19 and 21 mm of fungi were much higher compared to the past three concentrations. Notably, these values were slightly lower than standard antibiotics, which typically show inhibition zones of 22 mm and 23 mm for the respective organisms. These results align with reported findings for similar complexes^63,64^, reinforcing the compound’s potential in antibacterial applications.

The determination of Minimum bactericidal and fungicidal Concentration (MBC, MFC) (Table S7) highlights the compound’s potent bactericidal and fungicidal activity against gram-positive bacteria. This selective activity was previously observed for mixed ligand-type Schiff base complexes of nickel, where metal ions enhanced antibacterial activity by reducing compound polarity and increasing lipophilicity^65,66^. The observed minimum MIC and MBC values against *Staphylococcus aureus*,* candida albicans*, and *candida tropicalis* fungi strongly suggest the compound’s ability to inhibit microbial growth at low concentrations, underscoring its therapeutic potential^39^. Comparison with literature data emphasized the compound’s promising pharmacological properties, with the azomethine linkage playing a crucial role in its biological activity^56^. Overall, these results highlight its potential as a therapeutic agent, warranting additional research into its mechanism of action and clinical uses.

### Molecular docking study

**Fig. 7:**
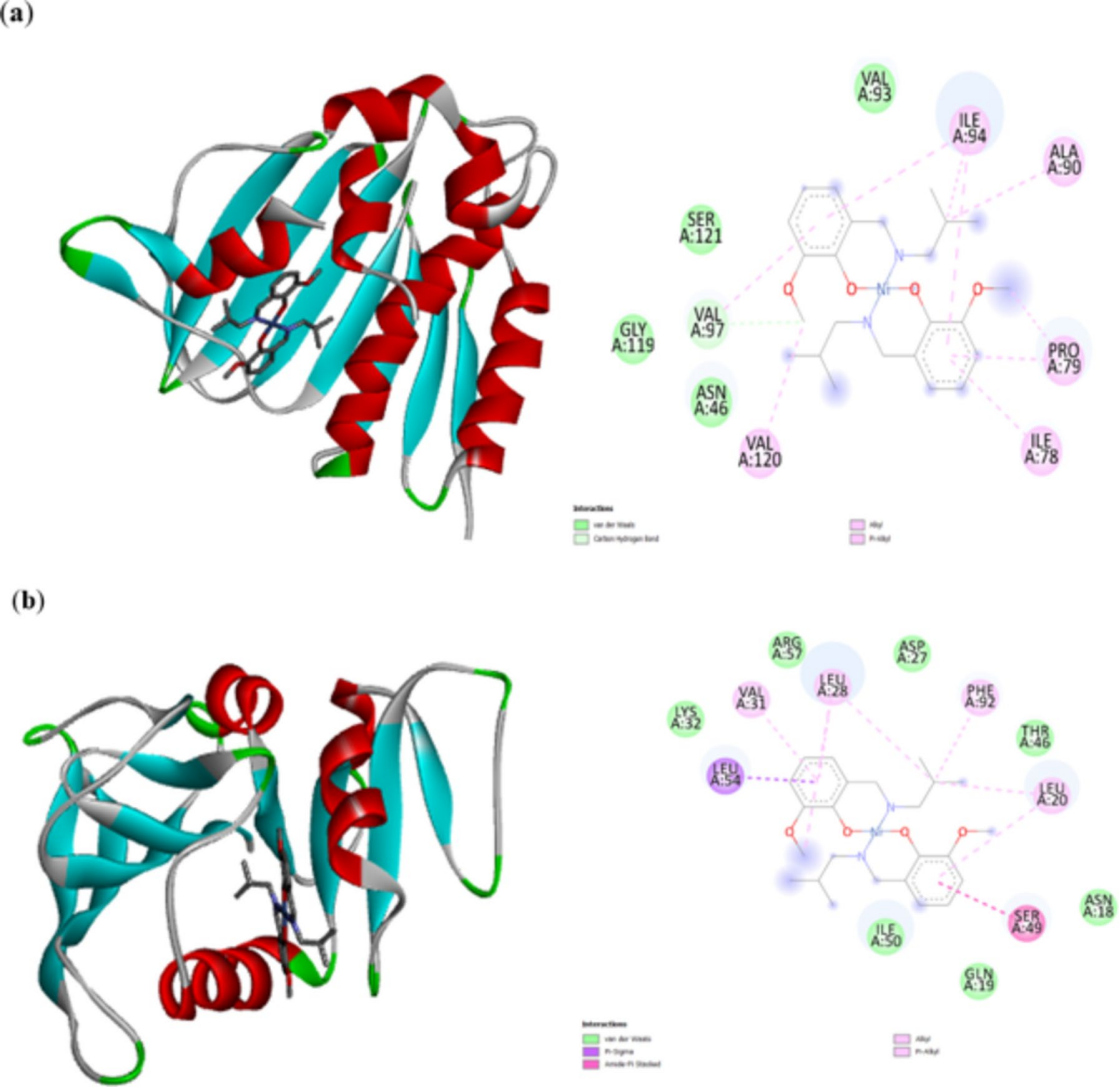

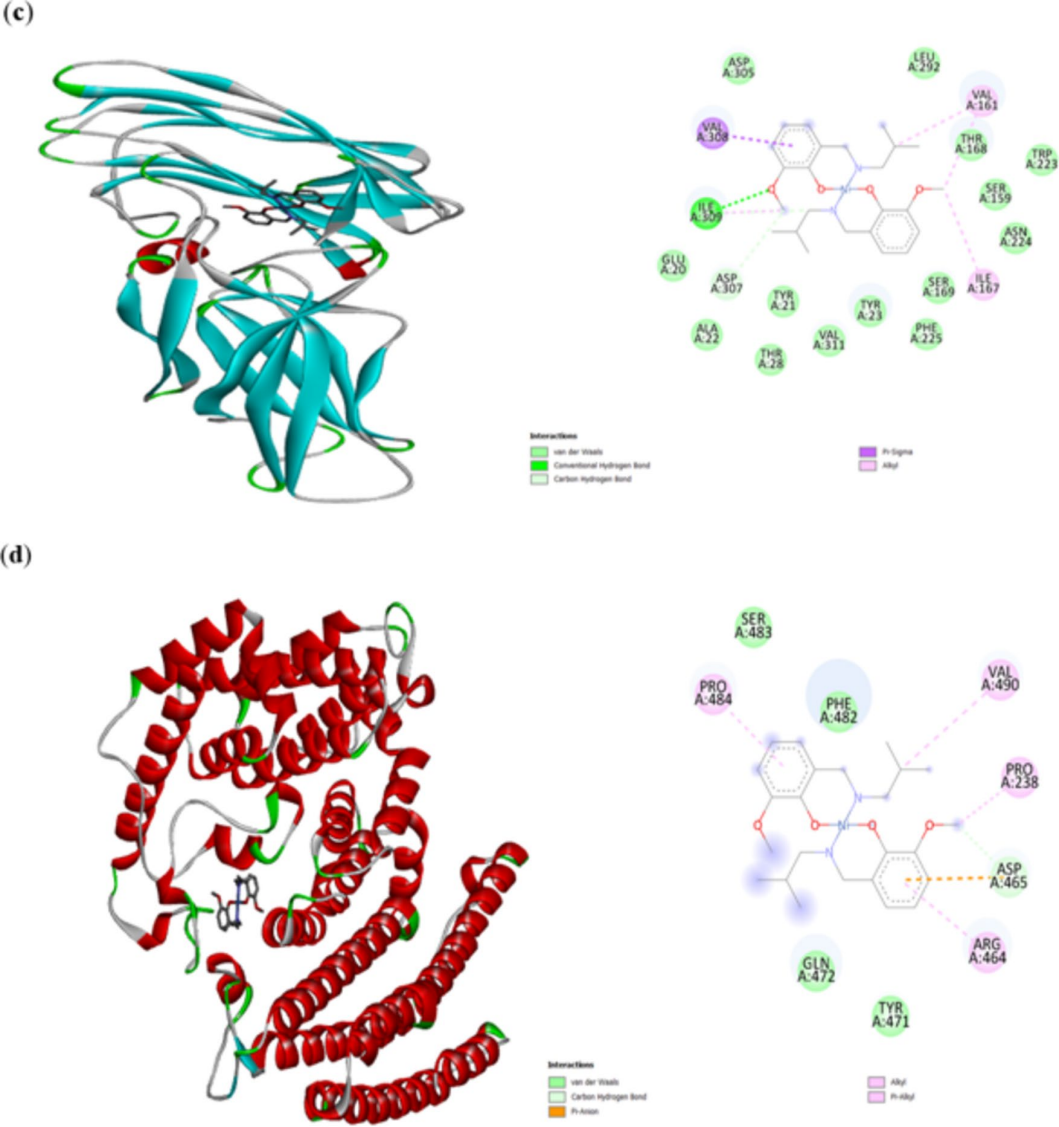
3D and 2D representations of the binding interactions of **2** against, (**a**) *E. coli* (PDB: 6F86), (**b**) *S. aureus* (PDB: 2w9h), (**c**) *C. albicans* (PDB: 2YLH), (**d**) *C. tropicalis* (PDB: 8BH9) was generated using AutoDock Tool (ADT) version 1.5.6 and AutoDock version 4.2.6.

The molecular docking results show the interaction of the ligand (complex **2**) and the receptor and provide quantitative binding energy. The effectiveness of the interaction is validated by the binding energy, type of interaction, and inhibition constant (k_i_)^58^. Lowest energy docked poses of **2** with different protein targets such as *Escherichia coli* (DNA Gyrase B), *Staphylococcus aureus* (Dihydrofolate Reductase), *Candida albicans* (Agglutinin-Like Als9 protein), and *Candida tropicalis* (PCIF1_WW domain-containing protein) are depicted in Fig. 7a-d and their corresponding docking parameters such as binding energy, inhibition constant and intermolecular energy are listed in Table 1.

**Table 1:** The obtained docking parameters of **2** docked with bacteria and fungi-associated targeted proteins on their rank calculated by Autodock.

S. no	PDBID	Binding energy	Inhibition constant	Intermolecular energy
		(kcal.mol^-1^)	(μM)	(kcal.mol^-1^)
1	Bacterium: Escherichia coli Target: DNA Gyrase B (PDB ID: 6F86)	− 6.51	17.05	− 6.51
2	Bacterium: Staphylococcus aureus Target: Dihydrofolate Reductase (PDB ID: 2W9H)	− 8.62	0.483	− 8.62
3	Fungus: Candida albicans Target: Agglutinin-like Als9 protein (PDB ID: 2Y7L)	− 7.42	3.64	− 7.42
4	Fungus: Candida tropicalis Target: PCIF1WW domain-containing protein (PDB ID: 8BH9)	6.05	36.7	− 6.05

Complex **2** has a moderate interaction with the binding affinity of -6.51 kcal.mol^− 1^ against the protein target DNA gyrase B of *E.coli* (Fig. 7a). At the same time, it exhibited a strong binding affinity of -8.62 against Dihydrofolate Reductase of *S.aureus* (Fig. 7b) with a low inhibition constant of 0.483 µM indicating strong interaction. The negative intermolecular energy suggests a stable complex is formed between the drug and the protein. The predominant interactions in both targets were hydrophobic. In the case of the fungal targets, the complex displayed better binding affinity (-7.42 k.cal.mol^− 1^) and inhibition of the agglutinin-like Als9 protein of *Candida albicans* as shown in Fig. 7c, than the PCIF1_WW domain-containing protein of *Candida tropicalis* (-6.05 k.cal.mol^− 1^) as shown in Fig. 7d. In the docking against *Candida albicans* a significant H-bond was observed. Complex **2** acted as a better anti-bacterial agent against *S. aureus* species.

The binding energy of a similar Schiff base Ni (II) complex with BSA was reported as -8.7 kcal.mol^− 1^^67^, closest to the binding affinity portrayed by the title complex against dihydrofolate reductase of *S. aureus*. Further, the binding energy of Ni (II) complex with a DNA Hexamer d(CGATCG)_2_ was found to be -7.82 kcal.mol^− 1^ and exhibited better anti-cancerous activity against human lung adenocarcinoma cell lines (notably higher efficiency than cisplatin)^68^. Another, Schiff base Ni (II) complex displayed a binding affinity of -6.14 kcal.mol^− 1^ towards EGFR Kinase and − 6.98 kcal.mol^− 1^ against Tyrosine Kinase^69^ and is close to the binding affinity portrayed by the title compound against DNA gyrase B of *E.Coli*. The Ni (II) metal encompassed with 2,3-bis((E) − 2 hydroxy-3- methoxy benzylidene)amino) maleonitrile exhibited the highest anti-oxidant activity and showed a binding energy of -8.1 kcal.mol^− 1^ with BSA^70^. Yet another Schiff base Ni (II) complex, incorporating the azo-ligand displayed a binding energy of − 8.37 kcal.mol^− 1^ towards the target receptor − 1hnj (*E.Coli* FabH-CoA) Complex^71^. To conclude, it is emphasized that the docking score and the nature of molecular interactions such as hydrogen bonding, non-covalent, hydrophobic, and π…π interactions between the target protein and the metal complexes indicate a significant antiproliferative activity^72^.

## Conclusion

This research culminated in successfully synthesizing and thoroughly characterizing a nickel complex **2**. Single-crystal X-ray diffraction analysis unraveled the square planar geometry of **2**. A comprehensive suite of spectroscopic techniques, including UV-Vis, FT-IR, ^1^H NMR, ^13^C NMR, and mass spectrometry, corroborated the proposed structure and coordination mode. The complex exhibited promising thermal stability up to 225 °C, and yielded a final residue of 10.3% at 450 ^o^C. The UV-Vis spectra showed two prominent peaks at 236 nm and 260 nm, suggesting the presence of chromophores such as aromatic rings and conjugated double bonds. The IR and NMR spectra confirmed the presence of azomethine group (-C = N) associated with microbial activity of schiff base compounds. Mass spectra confirmed the monomeric mass (*m/z* = 471) in its solid state. In the biological evaluation, the observed minimum values of MIC, MIB/MFC imply that a considerably lesser quantity (30–40 µg/mL) of the complex is sufficient to inhibit the growth of the microorganism indicating its potential for microbial activity. The DFT study yielded an energy gap of 3.46 eV between HOMO and LUMO. The Hirshfield surface analysis showed that the predominant factors influencing surface interactions were, H…H (66.5%) and C…H (22.9%). Docking studies have demonstrated that the complex exhibits a strong affinity for proteins, including PDB: 2W9H (Dihydrofolate Reductase of S. aureus), characterised by a high and favourable binding energy of -8.62 kcal.mol^− 1^, indicative of its potential biological activity.

## Electronic supplementary material

Below is the link to the electronic supplementary material.

Supplementary Material 1

Supplementary Material 2

Supplementary Material 3

## Data Availability

The datasets generated and/or analyzed during the current study are available in the CCDC repository, through http://www.ccdc.cam.ac.uk/conts/retrieving.htmL or CCDC No. 2320704.
