# Supplementary material for: Synthesis and structural insights of bis(2-methoxy-6-{[(2-methylpropyl)imino]methyl}phenolato) nickel (II) complex through DFT and docking investigations
Source: Sci Rep. 2025 Jan 11;15:1751. doi: 10.1038/s41598-025-85465-6 (PMC11724891; doi:10.1038/s41598-025-85465-6)
Supplement: Supplementary file 1 — Supplementary Material 1 [file 41598_2025_85465_MOESM1_ESM.pdf]

checkCIF (basic structural check) running

Checking for embedded fcf data in CIF ...
Found embedded fcf data in CIF. Extracting fcf data from uploaded CIF, please wait . . .

checkCIF/PLATON (basic structural check)

Structure factors have been supplied for datablock(s) shelx

THIS REPORT IS FOR GUIDANCE ONLY. IF USED AS PART OF A REVIEW PROCEDURE FOR PUBLICATION, IT SHOULD NOT REPLACE THE EXPERTISE OF AN EXPERIENCED CRYSTALLOGRAPHIC REFEREE.

No syntax errors found. CIF dictionary
Please wait while processing .... Interpreting this report

Structure factor report

Datablock: shelx

Bond precision:	C-C = 0.0050 A		Wavelength=0.71073
Cell:	a=6.2835(5)	b=17.4797(14)	c=21.2542(17)
	alpha=90	beta=93.779(3)	gamma=90
Temperature:	303 K		

	Calculated	Reported
Volume	2329.4(3)	2329.4(3)
Space group	P 21/n	P 21/n
Hall group	-P 2yn	-P 2yn
Moiety formula	C24 H32 N2 Ni O4	?
Sum formula	C24 H32 N2 Ni O4	C24 H32 N2 Ni O4
Mr	471.21	471.22
Dx,g cm-3	1.344	1.344
Z	4	4
Mu (mm-1)	0.865	0.865
F000	1000.0	1000.0
F000'	1001.72	
h,k,lmax	7,21,25	7,21,25
Nref	4439	4423
Tmin,Tmax	0.885,0.958	0.636,0.745
Tmin'	0.778	

Correction method= # Reported T Limits: Tmin=0.636 Tmax=0.745 AbsCorr = MULTI-SCAN

Data completeness= 0.996 Theta(max)= 25.682

R(reflections)= 0.0472(3014) wR2(reflections)= 0.0960(4423)

S = 1.073 Npar= 286

The following ALERTS were generated. Each ALERT has the format
test-name_ALERT_alert-type_alert-level.
Click on the hyperlinks for more details of the test.

Alert level C

PLAT242_ALERT_2_C Low 'MainMol' Ueq as Compared to Neighbors of C10 Check
PLAT242_ALERT_2_C Low 'MainMol' Ueq as Compared to Neighbors of C22 Check
PLAT906_ALERT_3_C Large K Value in the Analysis of Variance ...... 4.914 Check
PLAT910_ALERT_3_C Missing # of FCF Reflection(s) Below Theta(Min). 7 Note
PLAT911_ALERT_3_C Missing FCF Refl Between Thmin & STh/L= 0.600 3 Report

Alert level G

PLAT480_ALERT_4_G Long H...A H-Bond Reported H10 ..O3 . 2.61 Ang.
PLAT480_ALERT_4_G Long H...A H-Bond Reported H22 ..O1 . 2.63 Ang.
PLAT794_ALERT_5_G Tentative Bond Valency for Ni1 (III) . 2.74 Info
PLAT883_ALERT_1_G No Info/Value for _atom_sites_solution_primary . Please Do !
PLAT912_ALERT_4_G Missing # of FCF Reflections Above STh/L= 0.600 6 Note
PLAT978_ALERT_2_G Number C-C Bonds with Positive Residual Density. 2 Info

---

0 **ALERT level A** = Most likely a serious problem - resolve or explain
0 **ALERT level B** = A potentially serious problem, consider carefully
5 **ALERT level C** = Check. Ensure it is not caused by an omission or oversight
6 **ALERT level G** = General information/check it is not something unexpected

1 ALERT type 1 CIF construction/syntax error, inconsistent or missing data
3 ALERT type 2 Indicator that the structure model may be wrong or deficient
3 ALERT type 3 Indicator that the structure quality may be low
3 ALERT type 4 Improvement, methodology, query or suggestion
1 ALERT type 5 Informative message, check

It is advisable to attempt to resolve as many as possible of the alerts in all categories. Often the minor alerts point to easily fixed oversights, errors and omissions in your CIF or refinement strategy, so attention to these fine details can be worthwhile. In order to resolve some of the more serious problems it may be necessary to carry out additional measurements or structure refinements. However, the purpose of your study may justify the reported deviations and the more serious of these should normally be commented upon in the discussion or experimental section of a paper or in the "special_details" fields of the CIF. checkCIF was carefully designed to identify outliers and unusual parameters, but every test has its limitations and alerts that are not important in a particular case may appear. Conversely, the absence of alerts does not guarantee there are no aspects of the results needing attention. It is up to the individual to critically assess their own results and, if necessary, seek expert advice.

Publication of your CIF in IUCr journals

A basic structural check has been run on your CIF. These basic checks will be run on all CIFs submitted for publication in IUCr journals (*Acta Crystallographica*, *Journal of Applied Crystallography*, *Journal of Synchrotron Radiation*); however, if you intend to submit to *Acta Crystallographica Section C* or *E* or *IUCrData*, you should make sure that full publication checks are run on the final version of your CIF prior to submission.

Publication of your CIF in other journals

Please refer to the *Notes for Authors* of the relevant journal for any special instructions relating to CIF submission.

PLATON version of 06/07/2023; check.def file version of 30/06/2023

Datablock shelx - ellipsoid plot

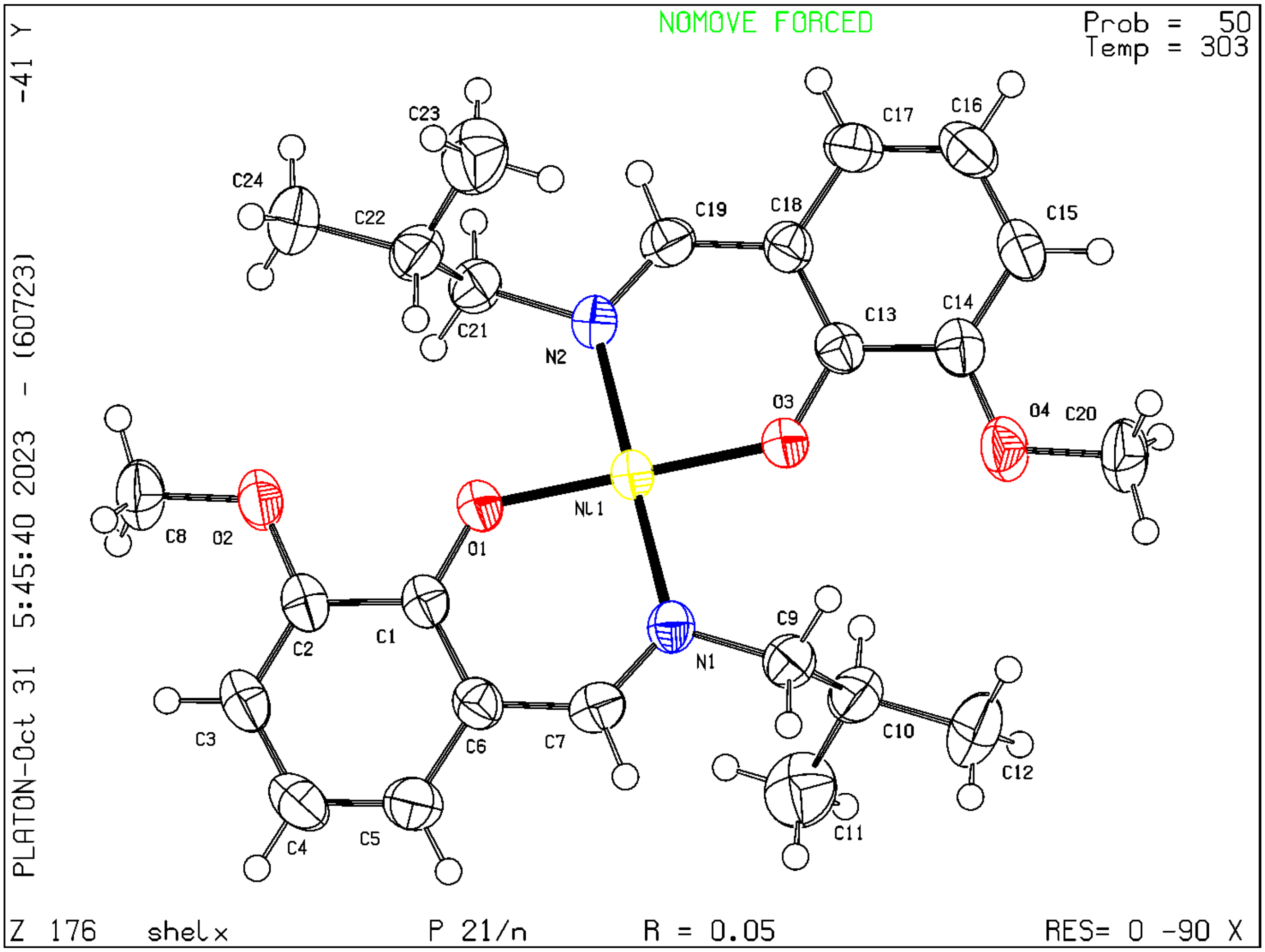

Download CIF editor (publCIF) from the IUCr
Download CIF editor (enCIFer) from the CCDC
Test a new CIF entry
