# Supplementary material for: Synthesis and structural insights of bis(2-methoxy-6-{[(2-methylpropyl)imino]methyl}phenolato) nickel (II) complex through DFT and docking investigations
Source: Sci Rep. 2025 Jan 11;15:1751. doi: 10.1038/s41598-025-85465-6 (PMC11724891; doi:10.1038/s41598-025-85465-6)
Supplement: Supplementary file 2 — Supplementary Material 2 [file 41598_2025_85465_MOESM2_ESM.pdf]

# Title

**Shobhana Krishnaswamy**

## Abstract

### Table 1

Experimental details

| | |
|---|---|
| Crystal data | |
| Chemical formula | $C_{24}H_{32}N_2NiO_4$ |
| $M_r$ | 471.22 |
| Crystal system, space group | Monoclinic, $P2_1/n$ |
| Temperature (K) | 303 |
| *a*, *b*, *c* (Å) | 6.2835 (5), 17.4797 (14), 21.2542 (17) |
| $\beta$ (°) | 93.779 (3) |
| $V$ (Å$^3$) | 2329.4 (3) |
| $Z$ | 4 |
| Radiation type | Mo $K\alpha$ |
| $\mu$ (mm$^{-1}$) | 0.87 |
| Crystal size (mm) | 0.29 × 0.12 × 0.05 |
| | |
| Data collection | |
| Diffractometer | Bruker D8 Venture Diffractometer |
| Absorption correction | Multi-scan<br>Krause, L., Herbst-Irmer, R., Sheldrick G.M. & Stalke D., J. Appl. Cryst. 48 (2015) 3-10. |
| $T_{min}$, $T_{max}$ | 0.636, 0.745 |
| No. of measured, independent and observed [$I > 2\sigma(I)$] reflections | 43485, 4423, 3014 |
| $R_{int}$ | 0.079 |
| $(\sin \theta/\lambda)_{max}$ (Å$^{-1}$) | 0.610 |
| | |
| Refinement | |
| $R[F^2 > 2\sigma(F^2)]$, $wR(F^2)$, $S$ | 0.047, 0.096, 1.07 |
| No. of reflections | 4423 |
| No. of parameters | 286 |
| H-atom treatment | H-atom parameters constrained |
| $\Delta\rho_{max}$, $\Delta\rho_{min}$ (e Å$^{-3}$) | 0.39, −0.31 |

Computer programs: *APEX4* (Bruker, 2021), *APEX4*/*SAINT* (Bruker, 2021), *SAINT*/*XPREP* (Bruker, 2021), *SHELXT* 2018/2 (Sheldrick, 2018), *SHELXL2019*/2 (Sheldrick, 2019), *WinGX*, *ORTEP-3* (Farrugia, 2012).

### Table 2

Hydrogen-bond geometry (Å, º)

| *D*—H···*A* | *D*—H | H···*A* | *D*···*A* | *D*—H···*A* |
|---|---|---|---|---|
| C10—H10···O3 | 0.98 | 2.61 | 3.126 (4) | 113 |

| | | | | |
|---|---|---|---|---|
| C22—H22···O1 | 0.98 | 2.63 | 3.138 (4) | 113 |

## Acknowledgements

## Funding information

## References

## Figure 1

# supporting information

## Title

### Computing details

Data collection: *APEX4* (Bruker, 2021); cell refinement: *APEX4*/*SAINT* (Bruker, 2021); data reduction: *SAINT*/*XPREP* (Bruker, 2021); program(s) used to solve structure: *SHELXT* 2018/2 (Sheldrick, 2018); program(s) used to refine structure: *SHELXL2019*/2 (Sheldrick, 2019), *WinGX*; molecular graphics: *ORTEP-3* (Farrugia, 2012).

### (shelx)

*Crystal data*

| | |
|---|---|
| $C_{24}H_{32}N_2NiO_4$ | $F(000) = 1000$ |
| $M_r = 471.22$ | $D_x = 1.344$ Mg m$^{-3}$ |
| Monoclinic, $P2_1/n$ | Mo $K\alpha$ radiation, $\lambda = 0.71073$ Å |
| $a = 6.2835$ (5) Å | Cell parameters from 8021 reflections |
| $b = 17.4797$ (14) Å | $\theta = 3.0–25.3°$ |
| $c = 21.2542$ (17) Å | $\mu = 0.87$ mm$^{-1}$ |
| $\beta = 93.779$ (3)° | $T = 303$ K |
| $V = 2329.4$ (3) Å$^3$ | Block, green |
| $Z = 4$ | 0.29 × 0.12 × 0.05 mm |

*Data collection*

| | |
|---|---|
| Bruker D8 Venture Diffractometer | 4423 independent reflections |
| Radiation source: fine focus sealed tube | 3014 reflections with $I > 2\sigma(I)$ |
| $\varphi$ and $\omega$ scans | $R_{int} = 0.079$ |
| Absorption correction: multi-scan Krause, L., Herbst-Irmer, R., Sheldrick G.M. & Stalke D., J. Appl. Cryst. 48 (2015) 3-10. | $\theta_{max} = 25.7°$, $\theta_{min} = 3.3°$ |
| | $h = -6\rightarrow7$ |
| | $k = -21\rightarrow21$ |
| $T_{min} = 0.636$, $T_{max} = 0.745$ | $l = -25\rightarrow25$ |
| 43485 measured reflections | |

*Refinement*

| | |
|---|---|
| Refinement on $F^2$ | Hydrogen site location: inferred from neighbouring sites |
| Least-squares matrix: full | H-atom parameters constrained |
| $R[F^2 > 2\sigma(F^2)] = 0.047$ | $w = 1/[\sigma^2(F_o^2) + (0.0188P)^2 + 2.7467P]$ where $P = (F_o^2 + 2F_c^2)/3$ |
| $wR(F^2) = 0.096$ | $(\Delta/\sigma)_{max} = 0.001$ |
| $S = 1.07$ | $\Delta\rho_{max} = 0.39$ e Å$^{-3}$ |
| 4423 reflections | $\Delta\rho_{min} = -0.31$ e Å$^{-3}$ |
| 286 parameters | |
| 0 restraints | |

*Special details*

*Geometry*. All e.s.d.'s (except the e.s.d. in the dihedral angle between two l.s. planes) are estimated using the full covariance matrix. The cell e.s.d.'s are taken into account individually in the estimation of e.s.d.'s in distances, angles and torsion angles; correlations between e.s.d.'s in cell parameters are only used when they are defined by crystal symmetry. An approximate (isotropic) treatment of cell e.s.d.'s is used for estimating e.s.d.'s involving l.s. planes.

*Fractional atomic coordinates and isotropic or equivalent isotropic displacement parameters (Å$^2$)*

| | $x$ | $y$ | $z$ | $U_{iso}$*/$U_{eq}$ |
|---|---|---|---|---|
| Ni1 | 0.49697 (8) | 0.66443 (2) | 0.25183 (2) | 0.03225 (12) |

| | | | | |
|---|---|---|---|---|
| C1 | 0.7815 (5) | 0.76319 (17) | 0.19047 (15) | 0.0341 (7) |
| C2 | 0.9742 (5) | 0.80743 (17) | 0.19487 (16) | 0.0370 (8) |
| C3 | 1.0423 (6) | 0.84376 (18) | 0.14306 (17) | 0.0453 (9) |
| H3 | 1.167669 | 0.872155 | 0.146611 | 0.054* |
| C4 | 0.9269 (7) | 0.8390 (2) | 0.08484 (18) | 0.0540 (10) |
| H4 | 0.976028 | 0.863902 | 0.049952 | 0.065* |
| C5 | 0.7426 (6) | 0.7980 (2) | 0.07897 (17) | 0.0507 (9) |
| H5 | 0.666090 | 0.795063 | 0.040064 | 0.061* |
| C6 | 0.6666 (5) | 0.75984 (18) | 0.13180 (15) | 0.0381 (8) |
| C7 | 0.4718 (5) | 0.71785 (18) | 0.12445 (15) | 0.0392 (8) |
| H7 | 0.398854 | 0.721039 | 0.085027 | 0.047* |
| C8 | 1.2618 (6) | 0.8546 (2) | 0.26158 (19) | 0.0575 (10) |
| H8A | 1.368306 | 0.834790 | 0.235600 | 0.086* |
| H8B | 1.314874 | 0.853418 | 0.304966 | 0.086* |
| H8C | 1.228750 | 0.906415 | 0.249482 | 0.086* |
| C9 | 0.1854 (5) | 0.6372 (2) | 0.14182 (16) | 0.0419 (8) |
| H9A | 0.121392 | 0.666478 | 0.106710 | 0.050* |
| H9B | 0.085733 | 0.636840 | 0.174773 | 0.050* |
| C10 | 0.2198 (6) | 0.5554 (2) | 0.12018 (17) | 0.0518 (10) |
| H10 | 0.272902 | 0.525097 | 0.156714 | 0.062* |
| C11 | 0.3781 (7) | 0.5489 (2) | 0.0697 (2) | 0.0734 (13) |
| H11A | 0.326705 | 0.577306 | 0.033105 | 0.110* |
| H11B | 0.394555 | 0.496104 | 0.058509 | 0.110* |
| H11C | 0.513314 | 0.569164 | 0.085373 | 0.110* |
| C12 | 0.0044 (8) | 0.5230 (3) | 0.0959 (2) | 0.0973 (19) |
| H12A | −0.095601 | 0.528032 | 0.127907 | 0.146* |
| H12B | 0.020184 | 0.469872 | 0.085600 | 0.146* |
| H12C | −0.047020 | 0.550558 | 0.058913 | 0.146* |
| C13 | 0.2146 (5) | 0.56416 (17) | 0.31303 (15) | 0.0346 (7) |
| C14 | 0.0220 (5) | 0.52024 (18) | 0.30821 (16) | 0.0401 (8) |
| C15 | −0.0434 (6) | 0.48094 (19) | 0.35952 (17) | 0.0455 (9) |
| H15 | −0.167604 | 0.451916 | 0.355556 | 0.055* |
| C16 | 0.0739 (6) | 0.4840 (2) | 0.41737 (18) | 0.0537 (10) |
| H16 | 0.026985 | 0.457741 | 0.451882 | 0.064* |
| C17 | 0.2586 (6) | 0.5258 (2) | 0.42341 (16) | 0.0495 (9) |
| H17 | 0.336761 | 0.527714 | 0.462079 | 0.059* |
| C18 | 0.3310 (5) | 0.56605 (18) | 0.37130 (15) | 0.0379 (8) |
| C19 | 0.5243 (6) | 0.60961 (18) | 0.37889 (15) | 0.0404 (8) |
| H19 | 0.597991 | 0.606417 | 0.418216 | 0.048* |
| C20 | −0.2703 (6) | 0.4747 (2) | 0.2415 (2) | 0.0585 (11) |
| H20A | −0.233913 | 0.422276 | 0.250152 | 0.088* |
| H20B | −0.328288 | 0.479357 | 0.198700 | 0.088* |
| H20C | −0.374222 | 0.491424 | 0.269625 | 0.088* |
| C21 | 0.8017 (5) | 0.6945 (2) | 0.36294 (16) | 0.0444 (8) |
| H21A | 0.902902 | 0.696972 | 0.330468 | 0.053* |
| H21B | 0.868113 | 0.666394 | 0.398379 | 0.053* |
| C22 | 0.7512 (6) | 0.7754 (2) | 0.38415 (17) | 0.0495 (9) |
| H22 | 0.689503 | 0.803689 | 0.347514 | 0.059* |
| C23 | 0.5938 (8) | 0.7779 (3) | 0.4352 (2) | 0.0758 (13) |
| H23A | 0.460968 | 0.755672 | 0.419472 | 0.114* |
| H23B | 0.570708 | 0.830039 | 0.447139 | 0.114* |
| H23C | 0.649900 | 0.749497 | 0.471197 | 0.114* |

| | | | | |
|---|---|---|---|---|
| C24 | 0.9588 (8) | 0.8151 (3) | 0.4073 (2) | 0.0831 (16) |
| H24A | 0.933990 | 0.868958 | 0.411426 | 0.125* |
| H24B | 1.064788 | 0.806738 | 0.377462 | 0.125* |
| H24C | 1.008077 | 0.794341 | 0.447466 | 0.125* |
| N1 | 0.3835 (4) | 0.67572 (14) | 0.16584 (12) | 0.0345 (6) |
| N2 | 0.6089 (4) | 0.65264 (14) | 0.33807 (12) | 0.0355 (6) |
| O1 | 0.7208 (4) | 0.72961 (13) | 0.24107 (10) | 0.0416 (6) |
| O2 | 1.0740 (4) | 0.80901 (13) | 0.25368 (11) | 0.0486 (6) |
| O3 | 0.2716 (4) | 0.59953 (13) | 0.26288 (10) | 0.0428 (6) |
| O4 | −0.0841 (4) | 0.52091 (14) | 0.25020 (12) | 0.0506 (6) |

*Atomic displacement parameters (Å$^2$)*

| | $U^{11}$ | $U^{22}$ | $U^{33}$ | $U^{12}$ | $U^{13}$ | $U^{23}$ |
|---|---|---|---|---|---|---|
| Ni1 | 0.0287 (2) | 0.0323 (2) | 0.0358 (2) | −0.00384 (18) | 0.00290 (14) | 0.00030 (18) |
| C1 | 0.0342 (18) | 0.0274 (16) | 0.0415 (19) | 0.0010 (13) | 0.0079 (15) | −0.0022 (14) |
| C2 | 0.0332 (19) | 0.0288 (16) | 0.050 (2) | 0.0010 (14) | 0.0117 (16) | −0.0022 (15) |
| C3 | 0.043 (2) | 0.037 (2) | 0.058 (2) | −0.0049 (15) | 0.0189 (18) | −0.0013 (17) |
| C4 | 0.071 (3) | 0.043 (2) | 0.051 (2) | −0.008 (2) | 0.027 (2) | 0.0017 (18) |
| C5 | 0.065 (3) | 0.046 (2) | 0.041 (2) | −0.0052 (19) | 0.0067 (18) | 0.0004 (16) |
| C6 | 0.044 (2) | 0.0319 (17) | 0.0397 (19) | −0.0020 (15) | 0.0093 (15) | −0.0017 (14) |
| C7 | 0.046 (2) | 0.0353 (18) | 0.0363 (18) | 0.0048 (15) | −0.0014 (15) | −0.0015 (15) |
| C8 | 0.042 (2) | 0.058 (2) | 0.072 (3) | −0.0152 (18) | 0.0010 (19) | 0.002 (2) |
| C9 | 0.036 (2) | 0.047 (2) | 0.0420 (19) | −0.0001 (16) | −0.0013 (15) | 0.0035 (16) |
| C10 | 0.061 (3) | 0.048 (2) | 0.045 (2) | −0.0114 (18) | −0.0057 (18) | −0.0006 (17) |
| C11 | 0.081 (3) | 0.059 (3) | 0.082 (3) | 0.004 (2) | 0.013 (3) | −0.018 (2) |
| C12 | 0.095 (4) | 0.118 (4) | 0.078 (3) | −0.060 (3) | 0.002 (3) | −0.030 (3) |
| C13 | 0.0345 (19) | 0.0323 (17) | 0.0381 (18) | 0.0014 (14) | 0.0101 (14) | −0.0026 (14) |
| C14 | 0.037 (2) | 0.0358 (18) | 0.049 (2) | −0.0010 (15) | 0.0089 (16) | −0.0046 (16) |
| C15 | 0.045 (2) | 0.0369 (19) | 0.057 (2) | −0.0088 (16) | 0.0192 (18) | −0.0043 (17) |
| C16 | 0.069 (3) | 0.046 (2) | 0.049 (2) | −0.010 (2) | 0.023 (2) | 0.0027 (18) |
| C17 | 0.066 (3) | 0.047 (2) | 0.0353 (19) | −0.0074 (19) | 0.0073 (17) | 0.0005 (16) |
| C18 | 0.045 (2) | 0.0309 (17) | 0.0386 (19) | −0.0042 (15) | 0.0074 (15) | −0.0032 (14) |
| C19 | 0.049 (2) | 0.0366 (19) | 0.0346 (18) | 0.0002 (16) | −0.0016 (16) | −0.0015 (15) |
| C20 | 0.035 (2) | 0.062 (3) | 0.078 (3) | −0.0151 (18) | −0.0023 (19) | 0.003 (2) |
| C21 | 0.037 (2) | 0.053 (2) | 0.043 (2) | −0.0036 (16) | −0.0073 (15) | 0.0076 (17) |
| C22 | 0.059 (3) | 0.044 (2) | 0.044 (2) | −0.0093 (18) | −0.0064 (18) | −0.0011 (17) |
| C23 | 0.078 (3) | 0.067 (3) | 0.084 (3) | −0.009 (2) | 0.018 (3) | −0.027 (2) |
| C24 | 0.092 (4) | 0.090 (3) | 0.067 (3) | −0.053 (3) | 0.003 (3) | −0.011 (3) |
| N1 | 0.0354 (16) | 0.0292 (14) | 0.0390 (15) | −0.0008 (12) | 0.0029 (12) | −0.0017 (12) |
| N2 | 0.0315 (16) | 0.0349 (15) | 0.0400 (16) | −0.0032 (12) | 0.0012 (12) | −0.0009 (12) |
| O1 | 0.0363 (14) | 0.0497 (14) | 0.0386 (13) | −0.0125 (11) | 0.0023 (10) | 0.0051 (11) |
| O2 | 0.0411 (15) | 0.0502 (14) | 0.0542 (15) | −0.0171 (11) | −0.0001 (12) | 0.0085 (12) |
| O3 | 0.0405 (14) | 0.0516 (14) | 0.0360 (13) | −0.0143 (11) | 0.0000 (10) | 0.0042 (11) |
| O4 | 0.0320 (13) | 0.0581 (16) | 0.0612 (16) | −0.0107 (11) | −0.0001 (12) | 0.0085 (13) |

*Geometric parameters (Å, º)*

| | | | |
|---|---|---|---|
| Ni1—O1 | 1.836 (2) | C12—H12B | 0.9600 |
| Ni1—O3 | 1.842 (2) | C12—H12C | 0.9600 |
| Ni1—N1 | 1.928 (3) | C13—O3 | 1.303 (4) |
| Ni1—N2 | 1.930 (3) | C13—C18 | 1.397 (5) |

| | | | |
|---|---|---|---|
| C1—O1 | 1.304 (4) | C13—C14 | 1.431 (4) |
| C1—C6 | 1.400 (5) | C14—O4 | 1.363 (4) |
| C1—C2 | 1.434 (4) | C14—C15 | 1.374 (4) |
| C2—O2 | 1.361 (4) | C15—C16 | 1.392 (5) |
| C2—C3 | 1.364 (4) | C15—H15 | 0.9300 |
| C3—C4 | 1.395 (5) | C16—C17 | 1.370 (5) |
| C3—H3 | 0.9300 | C16—H16 | 0.9300 |
| C4—C5 | 1.360 (5) | C17—C18 | 1.412 (4) |
| C4—H4 | 0.9300 | C17—H17 | 0.9300 |
| C5—C6 | 1.416 (4) | C18—C19 | 1.434 (5) |
| C5—H5 | 0.9300 | C19—N2 | 1.289 (4) |
| C6—C7 | 1.427 (5) | C19—H19 | 0.9300 |
| C7—N1 | 1.300 (4) | C20—O4 | 1.423 (4) |
| C7—H7 | 0.9300 | C20—H20A | 0.9600 |
| C8—O2 | 1.425 (4) | C20—H20B | 0.9600 |
| C8—H8A | 0.9600 | C20—H20C | 0.9600 |
| C8—H8B | 0.9600 | C21—N2 | 1.482 (4) |
| C8—H8C | 0.9600 | C21—C22 | 1.525 (5) |
| C9—N1 | 1.476 (4) | C21—H21A | 0.9700 |
| C9—C10 | 1.522 (5) | C21—H21B | 0.9700 |
| C9—H9A | 0.9700 | C22—C23 | 1.516 (5) |
| C9—H9B | 0.9700 | C22—C24 | 1.529 (5) |
| C10—C11 | 1.515 (5) | C22—H22 | 0.9800 |
| C10—C12 | 1.526 (5) | C23—H23A | 0.9600 |
| C10—H10 | 0.9800 | C23—H23B | 0.9600 |
| C11—H11A | 0.9600 | C23—H23C | 0.9600 |
| C11—H11B | 0.9600 | C24—H24A | 0.9600 |
| C11—H11C | 0.9600 | C24—H24B | 0.9600 |
| C12—H12A | 0.9600 | C24—H24C | 0.9600 |

| | | | |
|---|---|---|---|
| O1—Ni1—O3 | 179.65 (12) | C18—C13—C14 | 117.8 (3) |
| O1—Ni1—N1 | 93.26 (10) | O4—C14—C15 | 124.6 (3) |
| O3—Ni1—N1 | 86.81 (10) | O4—C14—C13 | 114.9 (3) |
| O1—Ni1—N2 | 87.16 (10) | C15—C14—C13 | 120.5 (3) |
| O3—Ni1—N2 | 92.77 (10) | C14—C15—C16 | 120.8 (3) |
| N1—Ni1—N2 | 179.57 (13) | C14—C15—H15 | 119.6 |
| O1—C1—C6 | 123.6 (3) | C16—C15—H15 | 119.6 |
| O1—C1—C2 | 118.7 (3) | C17—C16—C15 | 120.0 (3) |
| C6—C1—C2 | 117.6 (3) | C17—C16—H16 | 120.0 |
| O2—C2—C3 | 125.6 (3) | C15—C16—H16 | 120.0 |
| O2—C2—C1 | 114.0 (3) | C16—C17—C18 | 120.4 (3) |
| C3—C2—C1 | 120.4 (3) | C16—C17—H17 | 119.8 |
| C2—C3—C4 | 121.1 (3) | C18—C17—H17 | 119.8 |
| C2—C3—H3 | 119.5 | C13—C18—C17 | 120.4 (3) |
| C4—C3—H3 | 119.5 | C13—C18—C19 | 120.2 (3) |
| C5—C4—C3 | 120.2 (3) | C17—C18—C19 | 119.4 (3) |
| C5—C4—H4 | 119.9 | N2—C19—C18 | 128.2 (3) |
| C3—C4—H4 | 119.9 | N2—C19—H19 | 115.9 |
| C4—C5—C6 | 120.3 (4) | C18—C19—H19 | 115.9 |
| C4—C5—H5 | 119.8 | O4—C20—H20A | 109.5 |
| C6—C5—H5 | 119.8 | O4—C20—H20B | 109.5 |
| C1—C6—C5 | 120.4 (3) | H20A—C20—H20B | 109.5 |

| | | | |
|---|---|---|---|
| C1—C6—C7 | 120.5 (3) | O4—C20—H20C | 109.5 |
| C5—C6—C7 | 119.1 (3) | H20A—C20—H20C | 109.5 |
| N1—C7—C6 | 128.2 (3) | H20B—C20—H20C | 109.5 |
| N1—C7—H7 | 115.9 | N2—C21—C22 | 112.5 (3) |
| C6—C7—H7 | 115.9 | N2—C21—H21A | 109.1 |
| O2—C8—H8A | 109.5 | C22—C21—H21A | 109.1 |
| O2—C8—H8B | 109.5 | N2—C21—H21B | 109.1 |
| H8A—C8—H8B | 109.5 | C22—C21—H21B | 109.1 |
| O2—C8—H8C | 109.5 | H21A—C21—H21B | 107.8 |
| H8A—C8—H8C | 109.5 | C23—C22—C21 | 113.3 (3) |
| H8B—C8—H8C | 109.5 | C23—C22—C24 | 109.9 (3) |
| N1—C9—C10 | 113.6 (3) | C21—C22—C24 | 109.0 (3) |
| N1—C9—H9A | 108.9 | C23—C22—H22 | 108.2 |
| C10—C9—H9A | 108.9 | C21—C22—H22 | 108.2 |
| N1—C9—H9B | 108.9 | C24—C22—H22 | 108.2 |
| C10—C9—H9B | 108.9 | C22—C23—H23A | 109.5 |
| H9A—C9—H9B | 107.7 | C22—C23—H23B | 109.5 |
| C11—C10—C9 | 113.4 (3) | H23A—C23—H23B | 109.5 |
| C11—C10—C12 | 110.0 (3) | C22—C23—H23C | 109.5 |
| C9—C10—C12 | 108.1 (4) | H23A—C23—H23C | 109.5 |
| C11—C10—H10 | 108.4 | H23B—C23—H23C | 109.5 |
| C9—C10—H10 | 108.4 | C22—C24—H24A | 109.5 |
| C12—C10—H10 | 108.4 | C22—C24—H24B | 109.5 |
| C10—C11—H11A | 109.5 | H24A—C24—H24B | 109.5 |
| C10—C11—H11B | 109.5 | C22—C24—H24C | 109.5 |
| H11A—C11—H11B | 109.5 | H24A—C24—H24C | 109.5 |
| C10—C11—H11C | 109.5 | H24B—C24—H24C | 109.5 |
| H11A—C11—H11C | 109.5 | C7—N1—C9 | 114.4 (3) |
| H11B—C11—H11C | 109.5 | C7—N1—Ni1 | 123.5 (2) |
| C10—C12—H12A | 109.5 | C9—N1—Ni1 | 122.1 (2) |
| C10—C12—H12B | 109.5 | C19—N2—C21 | 114.3 (3) |
| H12A—C12—H12B | 109.5 | C19—N2—Ni1 | 124.1 (2) |
| C10—C12—H12C | 109.5 | C21—N2—Ni1 | 121.6 (2) |
| H12A—C12—H12C | 109.5 | C1—O1—Ni1 | 130.6 (2) |
| H12B—C12—H12C | 109.5 | C2—O2—C8 | 116.6 (3) |
| O3—C13—C18 | 123.9 (3) | C13—O3—Ni1 | 130.7 (2) |
| O3—C13—C14 | 118.2 (3) | C14—O4—C20 | 117.5 (3) |

| | | | |
|---|---|---|---|
| O1—C1—C2—O2 | 0.0 (4) | O3—C13—C18—C19 | 0.9 (5) |
| C6—C1—C2—O2 | 178.8 (3) | C14—C13—C18—C19 | −179.5 (3) |
| O1—C1—C2—C3 | −179.6 (3) | C16—C17—C18—C13 | 0.4 (5) |
| C6—C1—C2—C3 | −0.9 (4) | C16—C17—C18—C19 | 179.8 (3) |
| O2—C2—C3—C4 | −179.5 (3) | C13—C18—C19—N2 | 3.1 (5) |
| C1—C2—C3—C4 | 0.1 (5) | C17—C18—C19—N2 | −176.2 (3) |
| C2—C3—C4—C5 | 0.3 (5) | N2—C21—C22—C23 | 58.9 (4) |
| C3—C4—C5—C6 | −0.1 (6) | N2—C21—C22—C24 | −178.4 (3) |
| O1—C1—C6—C5 | 179.8 (3) | C6—C7—N1—C9 | −177.4 (3) |
| C2—C1—C6—C5 | 1.1 (5) | C6—C7—N1—Ni1 | 3.2 (5) |
| O1—C1—C6—C7 | −0.3 (5) | C10—C9—N1—C7 | 96.0 (3) |
| C2—C1—C6—C7 | −179.0 (3) | C10—C9—N1—Ni1 | −84.6 (3) |
| C4—C5—C6—C1 | −0.7 (5) | C18—C19—N2—C21 | 175.5 (3) |
| C4—C5—C6—C7 | 179.4 (3) | C18—C19—N2—Ni1 | −3.6 (5) |

| | | | |
|---|---|---|---|
| C1—C6—C7—N1 | −4.4 (5) | C22—C21—N2—C19 | −95.6 (3) |
| C5—C6—C7—N1 | 175.5 (3) | C22—C21—N2—Ni1 | 83.5 (3) |
| N1—C9—C10—C11 | −57.0 (4) | C6—C1—O1—Ni1 | 5.9 (5) |
| N1—C9—C10—C12 | −179.3 (3) | C2—C1—O1—Ni1 | −175.5 (2) |
| O3—C13—C14—O4 | −0.6 (4) | N1—Ni1—O1—C1 | −5.6 (3) |
| C18—C13—C14—O4 | 179.9 (3) | N2—Ni1—O1—C1 | 174.4 (3) |
| O3—C13—C14—C15 | 179.0 (3) | C3—C2—O2—C8 | 2.1 (5) |
| C18—C13—C14—C15 | −0.5 (5) | C1—C2—O2—C8 | −177.5 (3) |
| O4—C14—C15—C16 | −179.4 (3) | C18—C13—O3—Ni1 | −4.1 (5) |
| C13—C14—C15—C16 | 1.1 (5) | C14—C13—O3—Ni1 | 176.4 (2) |
| C14—C15—C16—C17 | −0.9 (5) | N1—Ni1—O3—C13 | −177.0 (3) |
| C15—C16—C17—C18 | 0.1 (6) | N2—Ni1—O3—C13 | 3.0 (3) |
| O3—C13—C18—C17 | −179.7 (3) | C15—C14—O4—C20 | −3.1 (5) |
| C14—C13—C18—C17 | −0.2 (5) | C13—C14—O4—C20 | 176.5 (3) |

*Hydrogen-bond geometry (Å, º)*

| *D*—H···*A* | *D*—H | H···*A* | *D*···*A* | *D*—H···*A* |
|---|---|---|---|---|
| C10—H10···O3 | 0.98 | 2.61 | 3.126 (4) | 113 |
| C22—H22···O1 | 0.98 | 2.63 | 3.138 (4) | 113 |
