# Supplementary material for: Synthesis and structural insights of bis(2-methoxy-6-{[(2-methylpropyl)imino]methyl}phenolato) nickel (II) complex through DFT and docking investigations
Source: Sci Rep. 2025 Jan 11;15:1751. doi: 10.1038/s41598-025-85465-6 (PMC11724891; doi:10.1038/s41598-025-85465-6)
Supplement: Supplementary file 3 — Supplementary Material 3 [file 41598_2025_85465_MOESM3_ESM.pdf]

Supplementary Tables and Figures

Synthesis and structural insights of bis(2-methoxy-6-{[(2-methylpropyl)imino]methyl}phenolato) nickel (II) complex through DFT and docking Investigations

K. Jayachandiran[1], Sv. Esha[1], M. Savitha Lakshmi[1], S. Mahalakshmi[1], S. Arockiasamy[1,*]

[1]Chemistry Department, School of Advanced Sciences, Vellore Institute of Technology, Chennai-127, India

ORCID : 0000-0002-6993-2688 (S. Arockiasamy)

[*]Corresponding author: Dr. S. Arockiasamy

Vellore Institute of Technology-Chennai campus

Chennai-600127

e-mail: arockiasamy.s@vit.ac.in

Phone: +91-44-3993 1484, Fax: +91-44-3993 2555.

Tables

Table S1 Calculated wavelengths, oscillator strengths (f > 0.05), and main orbital transition contributions (≥ 30%) of absorption spectra of **2** in solution using TDDFT as well as their character and related experimental values.

	Wavelength (nm)			Assignment	
Complex	Experimental	Theoretical	f	Major contribution (%)	Character
**2**	246	249	0.34	HOMO-6 ->LUMO+2 (49)	LMCT
	266	263	0.51	HOMO-6 ->LUMO (46)	LMCT
				HOMO-3 ->LUMO+2 (32)	LMCT
	380	391	0.07	HOMO ->LUMO+1 (95)	MLCT

Table S2 Crystal data and structure refinement for **2**

Crystal data	
Empirical formula	$C_{24}H_{32}O_4N_2Ni$
Formula weight	471.22
Temperature (K)	303(2)
Wavelength (Å)	0.71073
Crystal system	Monoclinic
Space group	P 21/n
Absorption coefficient (mm^{-1})	0.865
F(000)	1000
Crystal size (mm^3)	0.290×0.118×0.050
Theta range for data collection (°)	3.327 to 25.682.
Index ranges (h,k,l)	$-6 \leq h \leq 7$, $-21 \leq k \leq 21$, $-25 \leq l \leq 25$
Reflections collected	43485
Independent reflections	4423 [R(int) = 0.0794]
Completeness to theta = 25.242°	99.8 %
Absorption correction	Semi-empirical from equivalents
Max. and min. transmission	0.7453 and 0.6362
Refinement method	Full-matrix least-squares on F^2
Data / restraints / parameters	4423 / 0 / 286
Goodness-of-fit on F^2	1.073
Final R indices [I>2sigma(I)]	R1 = 0.0472, wR2 = 0.0823
R indices (all data)	R1 = 0.0830, wR2 = 0.0960
Extinction coefficient	n/a
Largest diff. peak and hole (e.Å^{-3})	0.391 and -0.314

Table S3 Selected bond lengths [Å] and bond angel [°]

Bond length	[Å]	Bond angle	[°]
Ni(1)-N(2)	1.930(3)	O(1)-Ni(1)-O(3)	179.65(12)
Ni(1)-N(1)	1.928(3)	N(1)-Ni(1)-N(2)	179.57(13)
Ni(1)-O(3)	1.842(2)	O(1)-C(1)-C(6)	123.6(3)
Ni(1)-O(1)	1.836(2)	N(1)-C(7)-C(6)	128.2(3)
C(1)-O(1)	1.304(4)	O(1)-C(1)-C(2)	118.7(3)
C(7)-N(1)	1.300(4)	O(2)-C(2)-C(1)	114.0(3)
C(1)-C(6)	1.400(5)	O(2)-C(2)-C(3)	125.6(3)
C(2)-O(2)	1.361(4)	O(1)-Ni(1)-N(1)	93.26(10)
C(8)-O(2)	1.425(4)	O(3)-Ni(1)-N(1)	86.81(10)
C(9)-N(1)	1.300(4)	O(3)-Ni(1)-N(2)	92.77(10)
C(13)-O(3)	1.303(4)	O(1)-Ni(1)-N(2)	87.16(10)
C(14)-O(4)	1.363(4)	N(1)-C(9)-C(10)	113.6(3)
C(19)-N(2)	1.289(4)	O(3)-C(13)-C(18)	123.9(3)
C(20)-O(4)	1.423(4)	O(3)-C(13)-C(14)	118.2(3)
C(21)-N(2)	1.482(4)	C(18)-C(13)-C(14)	117.8(3)
		O(4)-C(14)-C(15)	124.6(3)
		O(4)-C(14)-C(13)	114.9(3)
		N(2)-C(19)-C(18)	128.2(3)
		O(4)-C(20)-H(20A)	109.5
		N(2)-C(21)-C(22)	112.5(3)
		C(7)-N(1)-Ni(1)	123.5(2)
		C(9)-N(1)-Ni(1)	122.1(2)
		C(19)-N(2)-C(21)	114.3(3)
		C(19)-N(2)-Ni(1)	124.1(2)
		C(21)-N(2)-Ni(1)	121.6(2)
		C(1)-O(1)-Ni(1)	130.6(2)
		C(14)-O(4)-C(20)	117.5(3)

Table S4 Quantum chemical descriptors of **2**

Parameters (eV)	
$E_{(HOMO)}$	-5.11
$E_{(LUMO)}$	-1.65
Energy gap (ΔE)	3.46
Ionisation potential (IP) $=-E_{(HOMO)}$	5.11
Electron affinity (EA) $=-E_{(LUMO)}$	1.65
Hardness(η) = $(E_{(LUMO)}-E_{(HOMO)})/2$	1.73
Softness(σ) = $1/2\eta$	0.29
Chemical potential (μ) = $-(I + A)/2$	-3.38
Electronegativity (χ) = $(I + A)/2$	3.38
Electrophilicity (ω) = $\chi^2/2\eta$	3.3
Nucleophilicity(ε) $=1/\omega$	0.3

Table S5 Antibacterial and antifungal activity of **2** using agar well diffusion method. PC: Positive control (Streptomycin for Bacteria and Imidazole for fungus); NC: Negative control

S.No	sample	Zone of inhibition (in mm)			
		Bacteria		Fungi	
		E.coli	*S.aureus*	*C.albicans*	*C.tropicalis*
1	NC (DMSO)	0	0	0	0
2	PC	21	21	21	21
3	Ni-complex	20	20	19	22

Table S6 Minimum Inhibitory Concentration (MIC). P* – Positive control: Streptomycin for bacteria, Imidazole for fungi.

S.No	Microorganism	Zone of Inhibition (in mm)				
		10 µg/mL	20 µg/mL	30 µg/mL	40 µg/mL	P*
1	E. coli	0	09	13	18	22
2	S. aureus	0	12	14	18	21
3	C. albicans	0	11	13	19	21
4	C. tropicalis	0	15	18	21	25

Table S7 Minimum bactericidal and fungicidal concentration (MBC, MFC). + Colonies, - No Colonies, NT: Not tested, MBC: Minimum bactericidal concentration, MFC: Minimum fungicidal concentration

S.No	Microorganism	Minimum bactericidal/ fungicidal concentration			
		10 µg/mL	20 µg/mL	30 µg/mL	40 µg/mL
1	E. coli	NT	+	+	(MBC)
2	S. aureus	NT	+	+	(MBC)
3	C. albicans	NT	+	(MFC)	-
4	C. tropicalis	NT	+	(MFC)	-

Figures

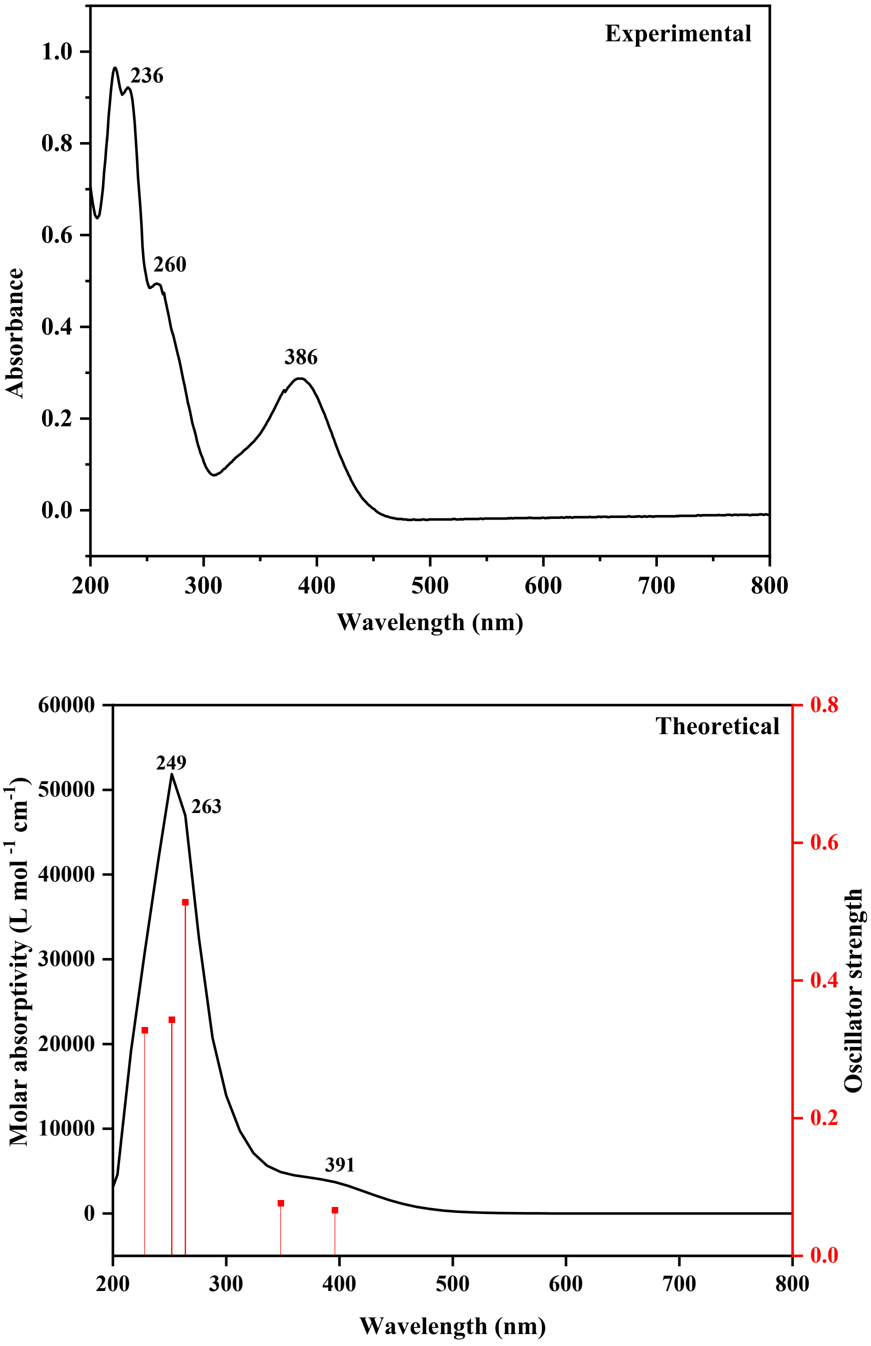

Figure S1. The experimental and theoretical UV-visible spectrum of **2.**

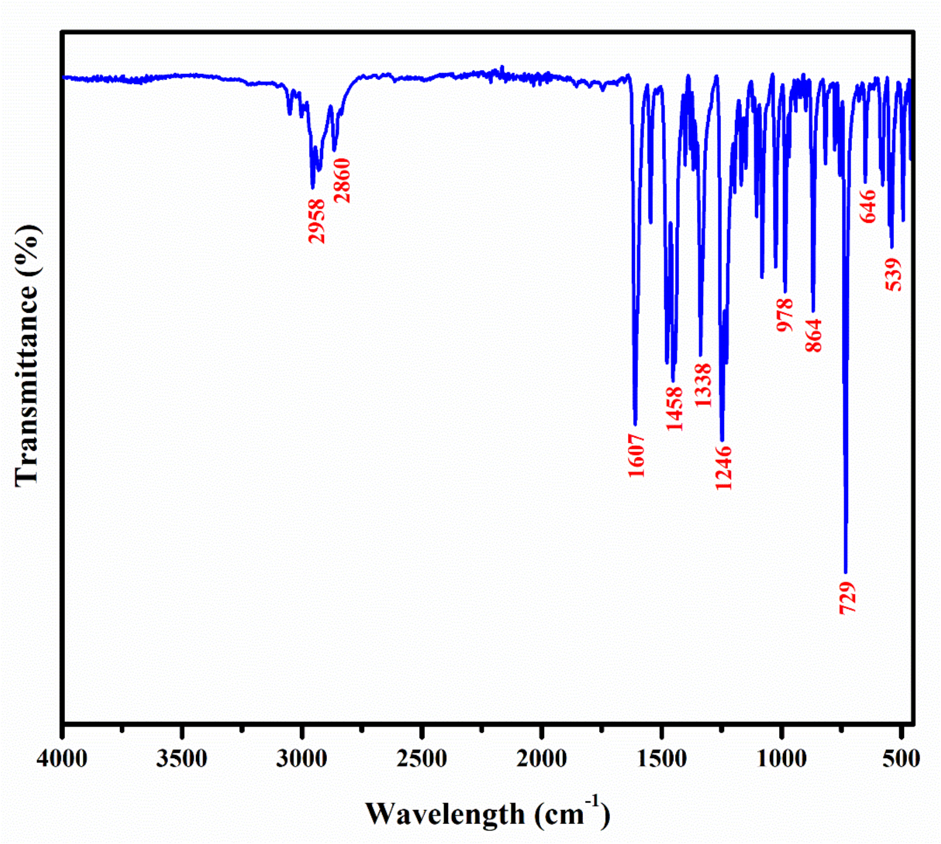

Figure S2. FTIR spectrum of **2**

(a)

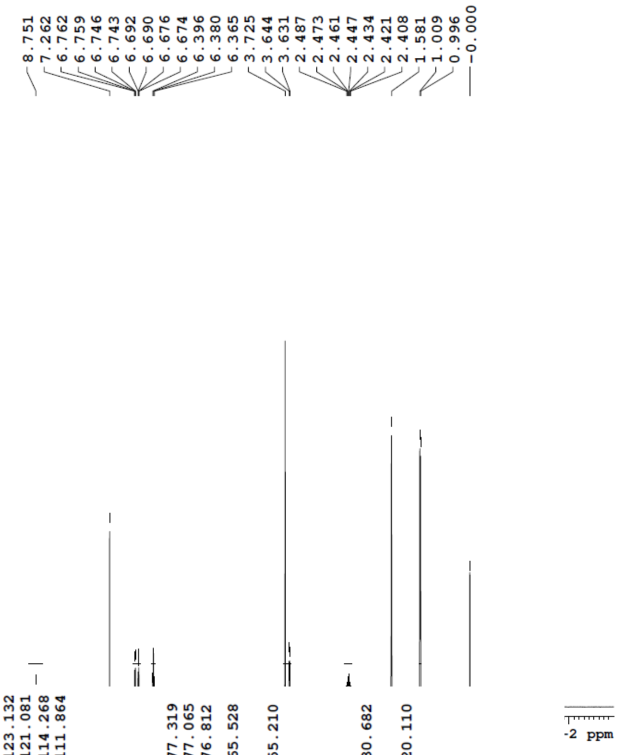

(b)

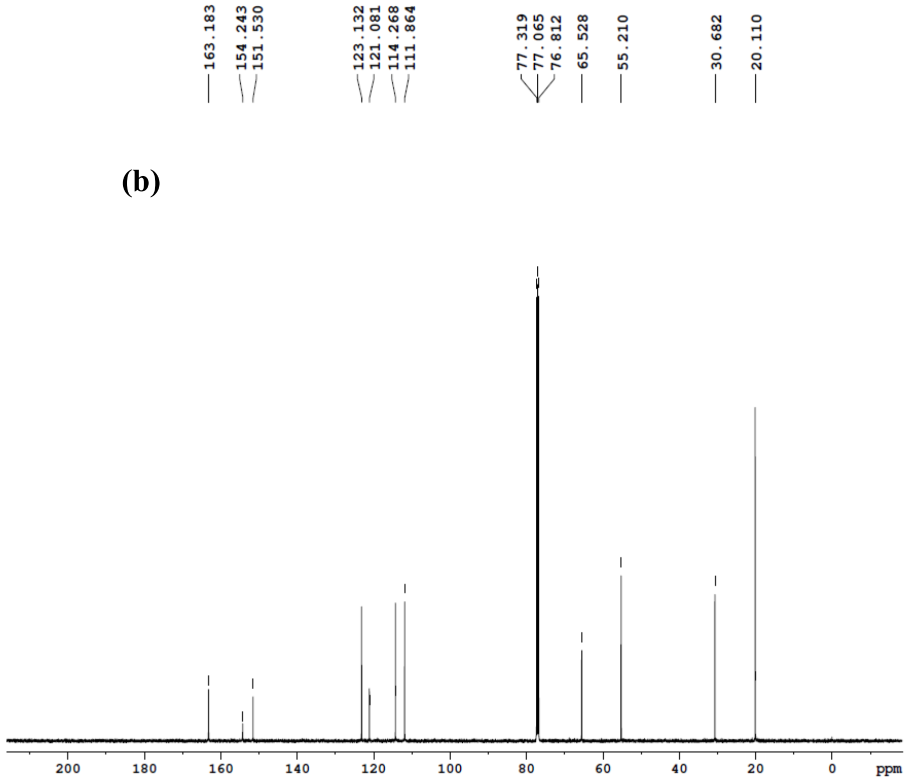

Figure S3. NMR spectra of **2** (a) ^{1}H NMR and (b) ^{13}C NMR.

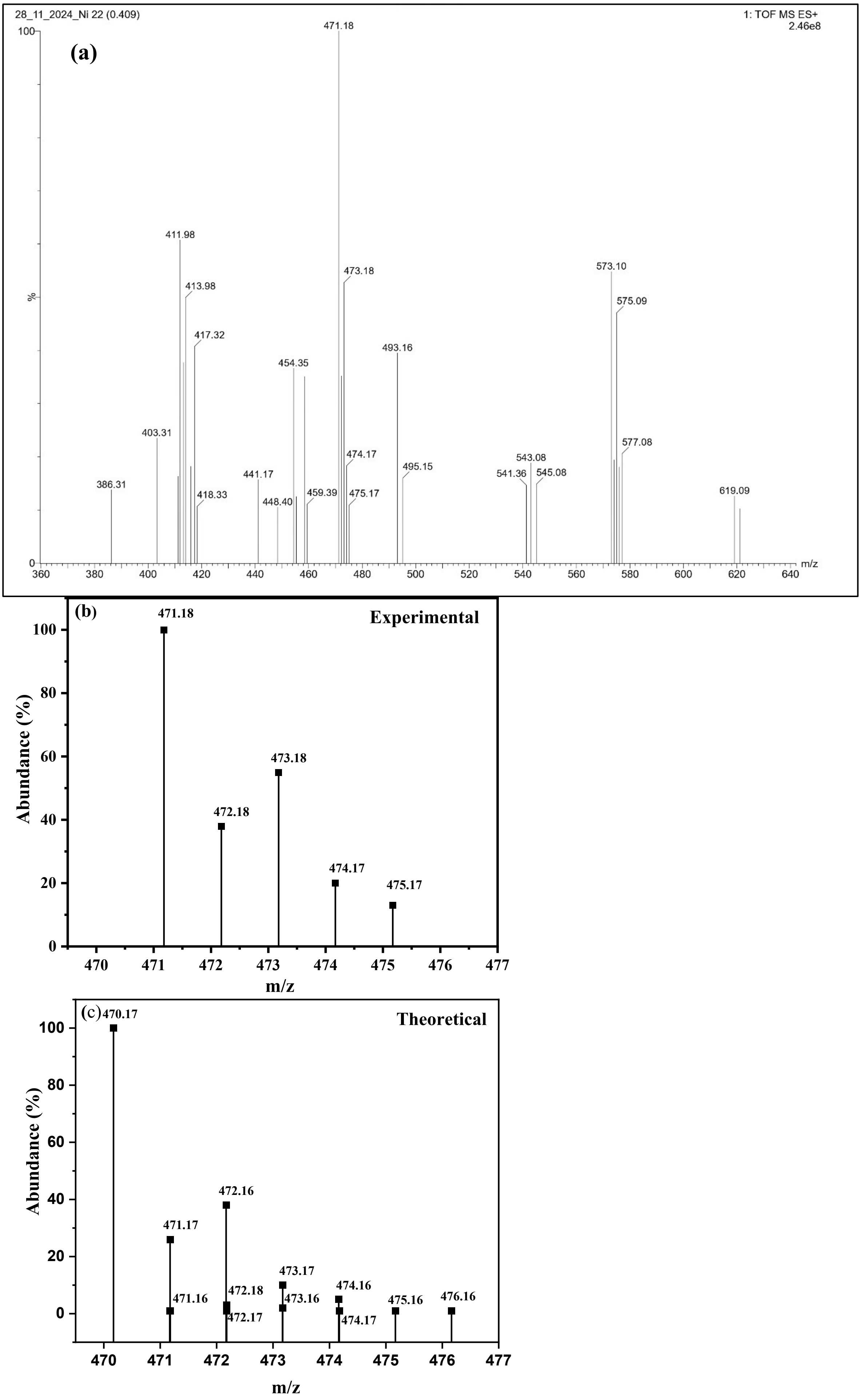
28_11_2024_Ni 22 (0.409)
1: TOF MS ES+
2.46e8
(a)
471.18
411.98
413.98
417.32
473.18
573.10
575.09
493.16
454.35
403.31
577.08
474.17
543.08
495.15
541.36
545.08
386.31
441.17
418.33
459.39
475.17
448.40
619.09
%
m/z
(b)
Experimental
471.18
473.18
472.18
474.17
475.17
Abundance (%)
m/z
(c)
470.17
Theoretical
472.16
471.17
473.17
472.18
474.16
471.16
473.16
475.16
476.16
472.17
474.17
Abundance (%)
m/z

Figure S4. Mass spectra, (a) Full spectrum, (b) experimental, and (c) theoretical isotopic distribution of complex **2**.

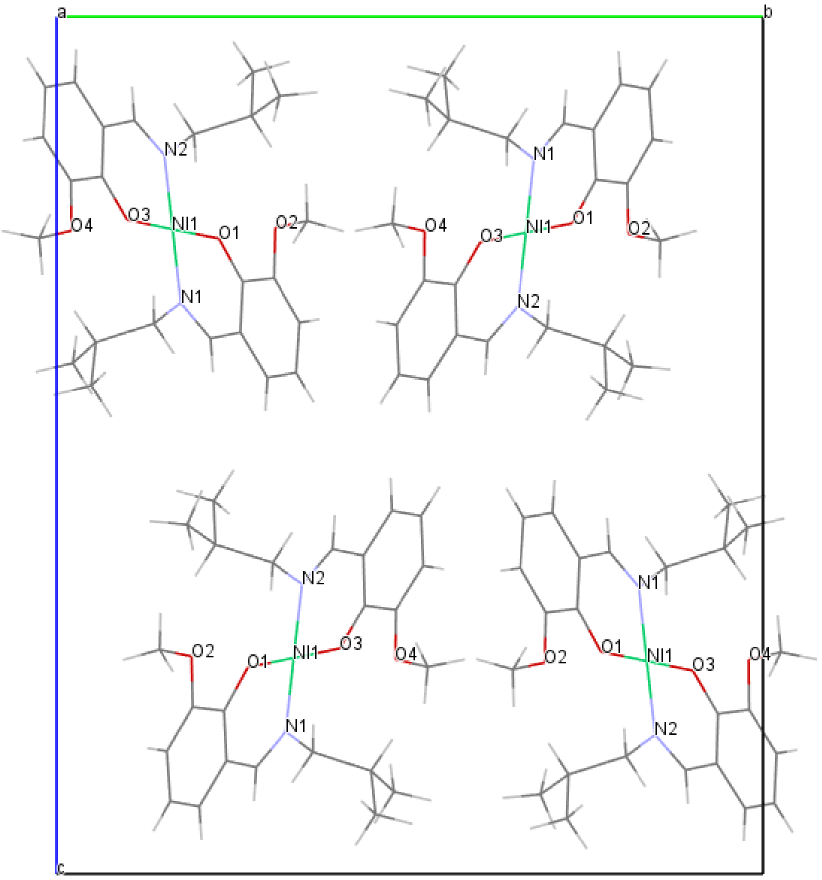

Figure S5. Packing pattern of **2** along the a-axis

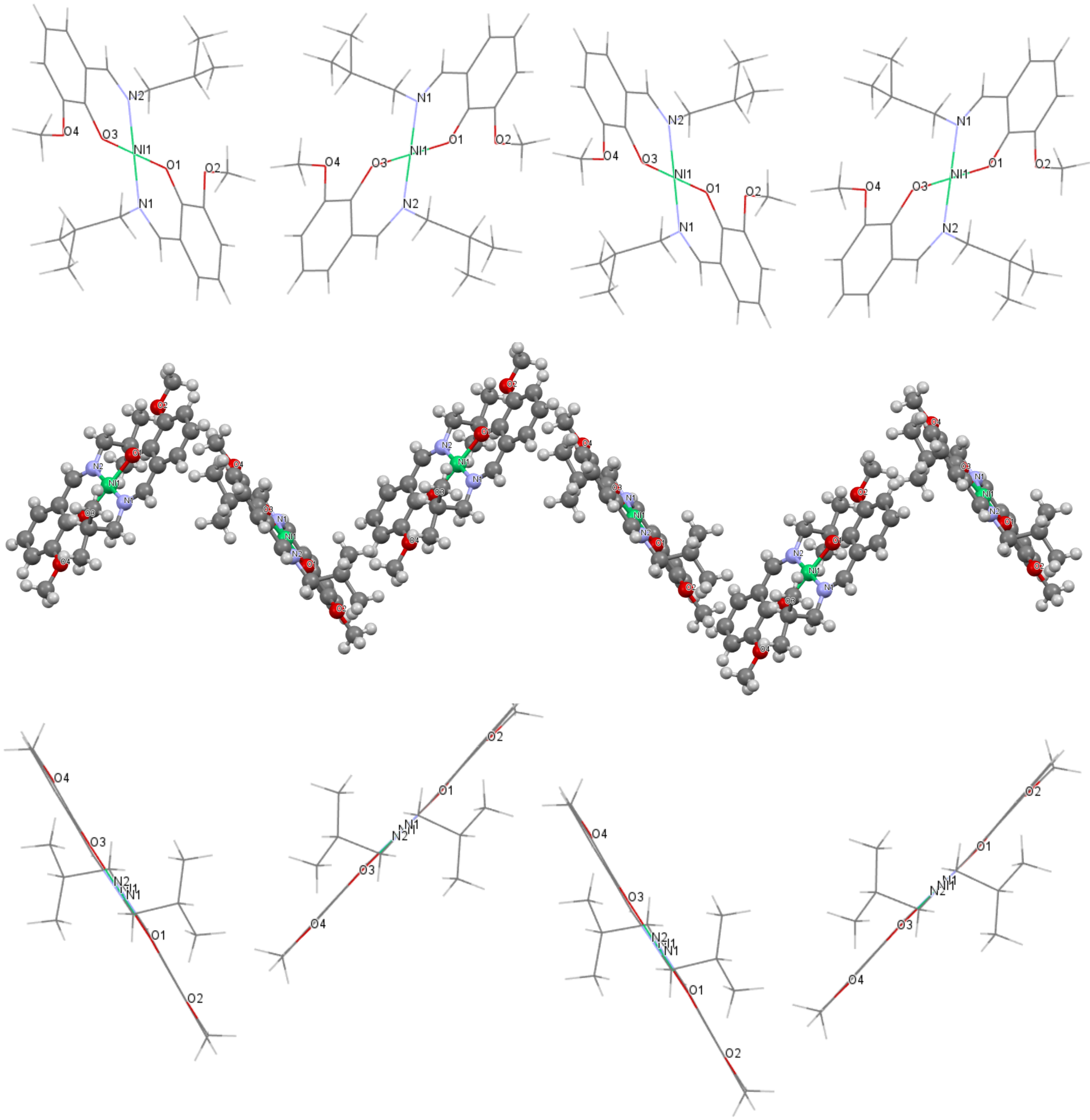

Figure S6. Antiparallel alignment of molecular planes

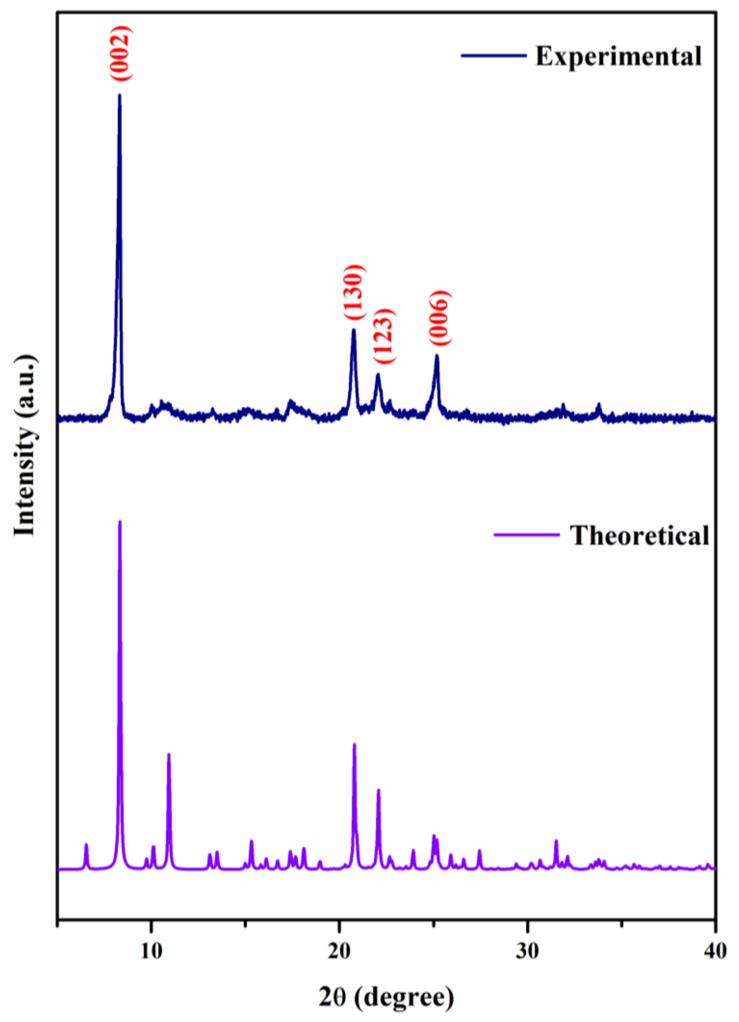

Figure S7. Powder X-ray diffraction pattern of Ni complex **2**

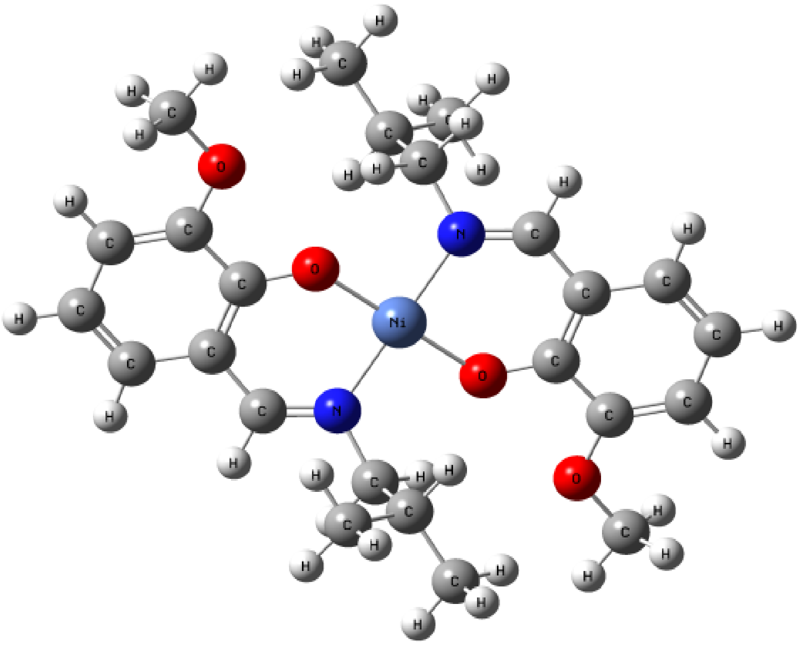

Figure S8. Optimized structure of the nickel complex.

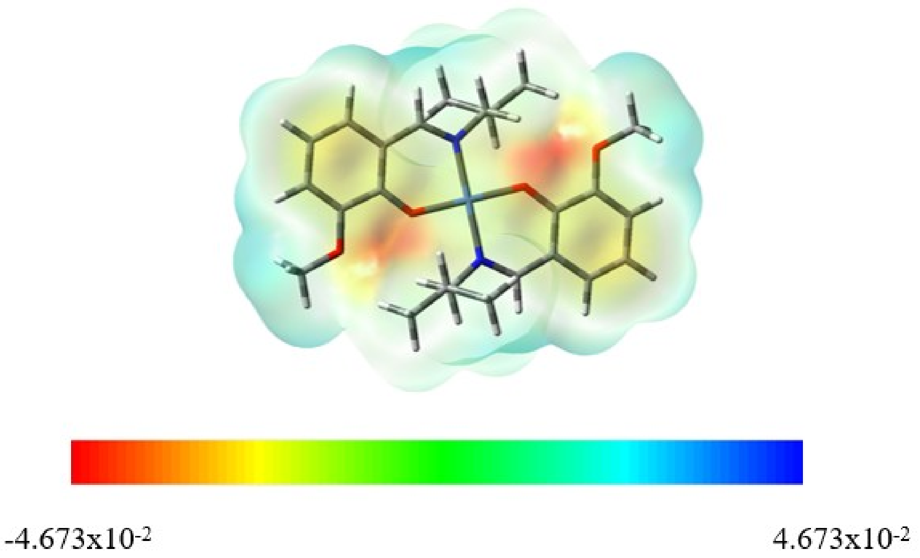

Figure S9. Mapped electrostatic potential surface of nickel complex generated using Gaussian software version 09.

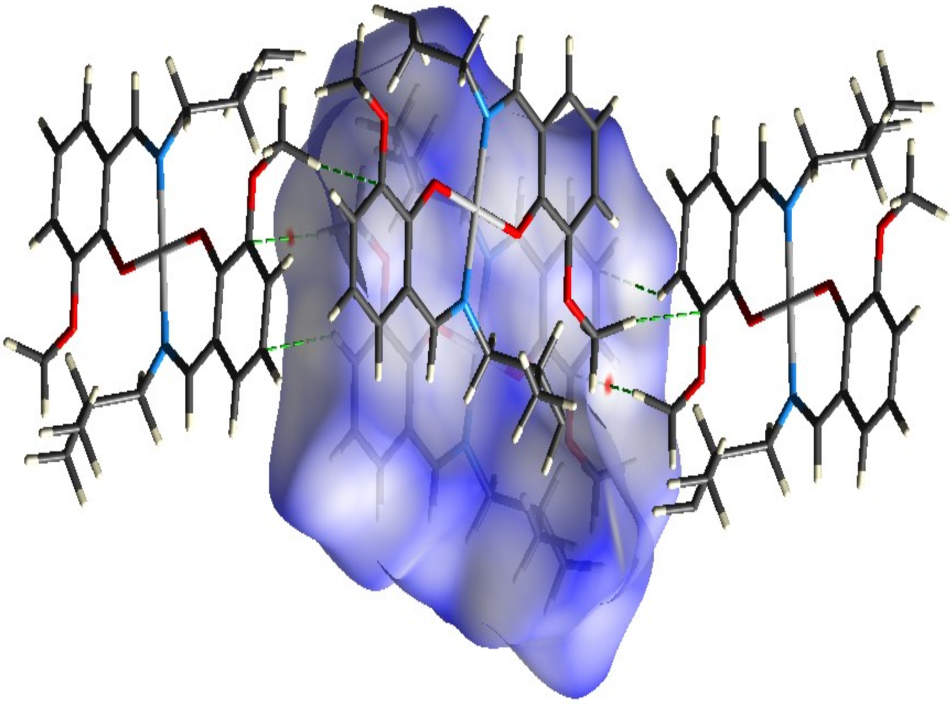

Figure S10. The d_{norm} surface of the nickel complex with intermolecular interactions was generated using Crystal Explorer version 21.5.
